# Polyphasic Analysis of a Middle Ages Coprolite Microbiota, Belgium

**DOI:** 10.1371/journal.pone.0088376

**Published:** 2014-02-28

**Authors:** Sandra Appelt, Fabrice Armougom, Matthieu Le Bailly, Catherine Robert, Michel Drancourt

**Affiliations:** 1 Unité de Recherche sur les Maladies Infectieuses Tropicales Emergentes, UM63, Centre national de la recherche scientifique 7278, IRD 198, Institut National de la Santé et de la Recherche Médicale 1095, Aix Marseille Université, Marseille, France; 2 Centre national de la recherche scientifique UMR 6249 Chrono-Environnement, Franche-Comté Université, Besançon, France; International Atomic Energy Agency, Austria

## Abstract

Paleomicrobiological investigations of a 14^th^-century coprolite found inside a barrel in Namur, Belgium were done using microscopy, a culture-dependent approach and metagenomics. Results were confirmed by *ad hoc* PCR – sequencing. Investigations yielded evidence for flora from ancient environment preserved inside the coprolite, indicated by microscopic observation of amoebal cysts, plant fibers, seeds, pollens and mold remains. Seventeen different bacterial species were cultured from the coprolite, mixing organisms known to originate from the environment and organisms known to be gut inhabitants. Metagenomic analyses yielded 107,470 reads, of which known sequences (31.9%) comprised 98.98% bacterial, 0.52% eukaryotic, 0.44% archaeal and 0.06% viral assigned reads. Most abundant bacterial phyla were *Proteobacteria*, *Gemmatimonadetes*, *Actinobacteria* and *Bacteroidetes*. The 16 S rRNA gene dataset yielded 132,000 trimmed reads and 673 Operational Taxonomic Units. Most abundant bacterial phyla observed in the 16 S rRNA gene dataset belonged to *Proteobacteria*, *Firmicutes*, *Actinobacteria* and *Chlamydia*. The Namur coprolite yielded typical gut microbiota inhabitants, intestinal parasites *Trichuris* and *Ascaris* and systemic pathogens *Bartonella* and *Bordetella*. This study adds knowledge to gut microbiota in medieval times.

## Introduction

Human paleomicrobiology, the quest for microbes in ancient specimens derived from humans, mainly relied on the investigations of old bone and dental pulp specimens [1]. Such investigations characterized past pathogens but did not provide data of ancient microbiota. In complement, investigating fossilized fecal material, i.e. coprolites, previously helped to gain knowledge on ancient human gut microbiota and intestinal parasites [2]–[6]. Currently, less than 20 coprolites and ancient colon content samples collected from six American and two European archeological sites have been investigated using large-scale sequencing and PCR-based analyses. These investigations yielded data about ancient gut microbiota, indicating that parts of the digestive flora were preserved in such specimens [2], [4], [5], [7]–[10]. Moreover, these studies enabled to compare dietary habits of ancient populations and their impact on human gut flora composition [4], [5], [10]. Analyzing differences in the composition of bacterial and fungal community associated with coprolites, supported the existence of two different pre-Columbian cultures in Indonesia [10]. Furthermore, a recent study indicated that coprolites exhibited more similarities between each other, and with stools from modern rural communities, than with stools coming from modern cosmopolitan communities. These findings support that modern lifestyle may participate to changes in the composition of the human gut flora [4].

In the present study, a further coprolite from a Middle Ages European site was investigated using a polyphasic approach in order to expand knowledge about gut microbiota in ancient Europe. Microscopic observations, culture and metagenomics (high-throughput sequencing and 16 S rRNA gene amplicon sequencing) were used to characterize the microbiota associated with the coprolite and to identify potential pathogens. Then, *ad hoc* suicide PCR amplifications were used for confirmation [11].

## Results

### Microscopic observations

**Figure 1:**
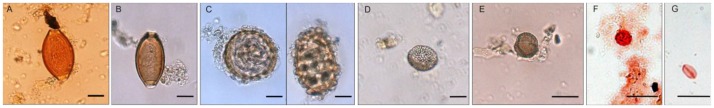
Microscopic observation of unstained and stained Namur coprolite (optical magnification: 100×). A) *Trichuris* spp. egg (ø 25.83 µm; length 43.65 µm); B) *Trichuris* spp. egg (ø 22.50 µm; length 41.10 µm); C) *Ascaris* spp. fertilized egg (ø 49.92 µm) and unfertilized egg (ø 43.16 µm; length 59.92 µm); D) pollen (ø 18.26 µm); E) suspected *Taenia* spp. egg (ø 14.65 µm); F) suspected *Acanthamoeba* spp. cyst; and G) seed remains. (The scale bar on the right indicates 20 µm).

In 1996, the exploration of an archeological Middle Ages site in Namur, Belgium yielded a closed barrel, such as those commonly used at that time as pits or latrines. The barrel was located at a depth of 3.80 m beneath the modern soil level and contained a 121.4 g, dark-brown, well preserved coprolite specimen. The specimen was attributed the number Z04F56 and was deposited into the laboratory of paleo- and parasitological studies of Champagne-Ardennes University, Reims, France until 2007. In 2007, the specimen was transferred to the laboratory for paleomicrobiology of Aix-Marseille University, Marseille, France for further investigations. After aseptically peeling of its external portion, the inner portion of the coprolite was re-suspended in sterile Page's amoeba saline medium (PAS) and microscopic observations revealed the presence of several eggs (Figure S1 in File S1). Thick-shelled and barrel-shaped eggs, with polar ‘plugs’ at the ends, 40–60 µm in length and 20–26 µm diameter, corresponded to the phenotypic description of *Trichuris* spp. eggs [12]. More precisely, broad eggs compatible with the pig-infecting *Trichuris suis* species and thinner eggs compatible with human-infecting *Trichuris trichiura* were observed (Figure 1A and B) [13]. Thick-shelled and brown eggs, corresponding to the description of *Ascaris* spp. eggs were also observed [12]. Among them, unfertilized elongated eggs (60 µm in length), fertilized round eggs (diameter between 40 and 50 µm), as well as eggs with embryos inside (Figure 1C) were found in the Namur coprolite. Microscopic observations also revealed the presence of suspected *Taenia* spp. eggs, plant fibers, pollens and mold remains (Figure 1D and 1E). Cysts, plant fibers and seeds stained red using Congo red (Figure 1F-G). The cysts measured 4.1 to 13.5 µm and matched with the description of amoeba cysts [14].

### Culture

After ten-day incubation at 30°C in the presence of negative controls, small colonies were visible in the aerobic and anaerobic layers of R2A and Schaedler broths. Additionally, a tiny film was observed on the surface of the R2A solid medium. After 5-7-day subculture, matrix-assisted laser desorption/ionization time-of-flight (MALDI-TOF) mass spectrometry performed as previously described [15] identified eight different bacterial species including *Paenibacillus macerans, Bacillus jeotgali*, *Staphylococcus pasteuri, Staphylococcus epidermidis*, *Staphylococcus cohnii*, *Micrococcus luteus*, *Pseudomonas geniculata* and *Stenotrophomonas maltophilia*. In addition *Bacillus horti* and *Clostridium magnum* were identified by 16 S rRNA gene sequencing [16] (Table S3 in File S1; Figure S2 in File S1). Furthermore, culturing the specimen in anaerobic and aerobic blood culture bottles in the presence of non-inoculated bottles (negative controls) yielded one *Rhodanobacter* sp. organism, one *Paenibacillus* sp. organism, *Paenibacillus macerans*, *Paenibacillus thiaminolyticus*, *Paenibacillus ehimensis*, *Staphylococcus arlettae*, *Propionibacterium acnes* and *Enterobacter cloacae* (Table S4 in File S1; Figure S2 in File S1).

### Metagenomics

**Figure 2:**
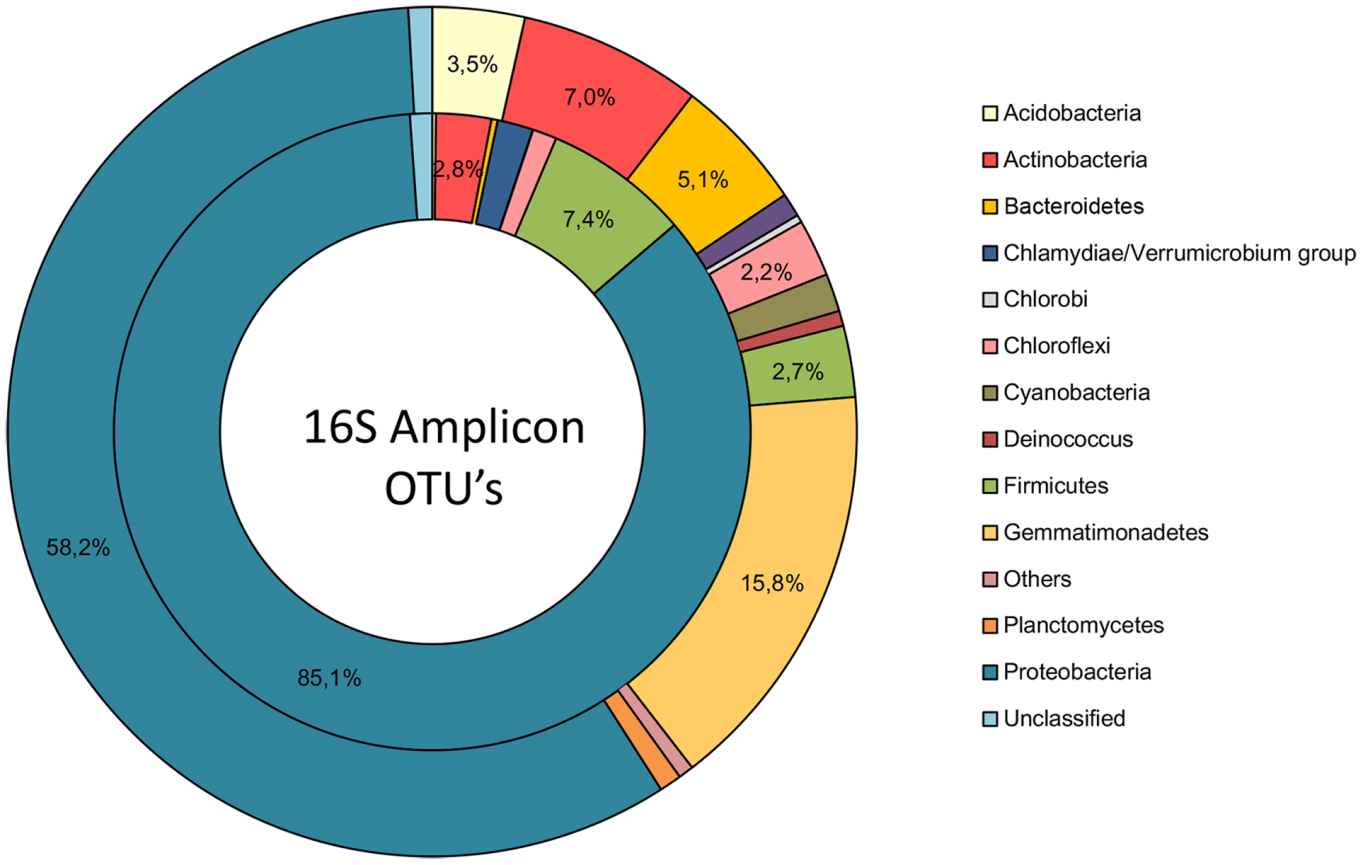
Composition of the Namur coprolite microbiota at the phylum level. The dataset obtained from high-throughput sequencing (external circle) and Operational Taxonomic Units (OTUs) assigned to the 16 S rRNA gene amplicon (internal circle) were used to identify bacterial phyla. Only phyla comprising more than 0.3% of the datasets are shown.

Acridine orange staining disclosed the presence of DNA in the coprolite, suggesting that molecular biological tools can be further applied to this specimen [10]. Accordingly, high-throughput sequencing yielded a total of 37.5 millions base pairs and 107,470 reads, with an average sequence length of 375 bp and a GC content between 65 and 70% (MG-RAST accession number 4479942.3). Taxonomic assignment of the reads was performed using a BLASTX comparison with the National Center for Biotechnology Information (NCBI) database, with stringent parameters as previously described [17], [18]. A significant similarity to known sequences was obtained for 31.9% of reads comprising 98.98% bacterial, 0.52% eukaryotic, 0.44% archaeal and 0.06% of viral reads. The most abundant bacterial phyla were *Proteobacteria* (58.12%), *Gemmatimonadetes*, (15.18%), *Actinobacteria* (6.96%) and *Bacteroidetes* (5.10%) (Figure 2). More precisely, the high-throughput sequencing dataset yielded *Gemmatimonas*, *Rhizobium*, *Streptomyces* and *Burkholderia* known as environmental bacteria; as well as *Corynebacterium, Cytophaga, Enterobacter, Prevotella*, *Ruminococcus*, *Aeromonas*, *Escherichia*, *Lactobacillus* and *Bacteroides* known as members of mammal gut microbiota (Table S1 in File S1 ). In particular, contig reconstruction and annotation identified contigs belonging to human gut *Bacteroides* species, including *Bacteroides finegoldii*, *Bacteroides vulgatus*, *Bacteroides coprocola* along with *Bacteroides coprosuis* belonging to pig gut microbiota (Table S2A in File S1).

**Figure 3:**
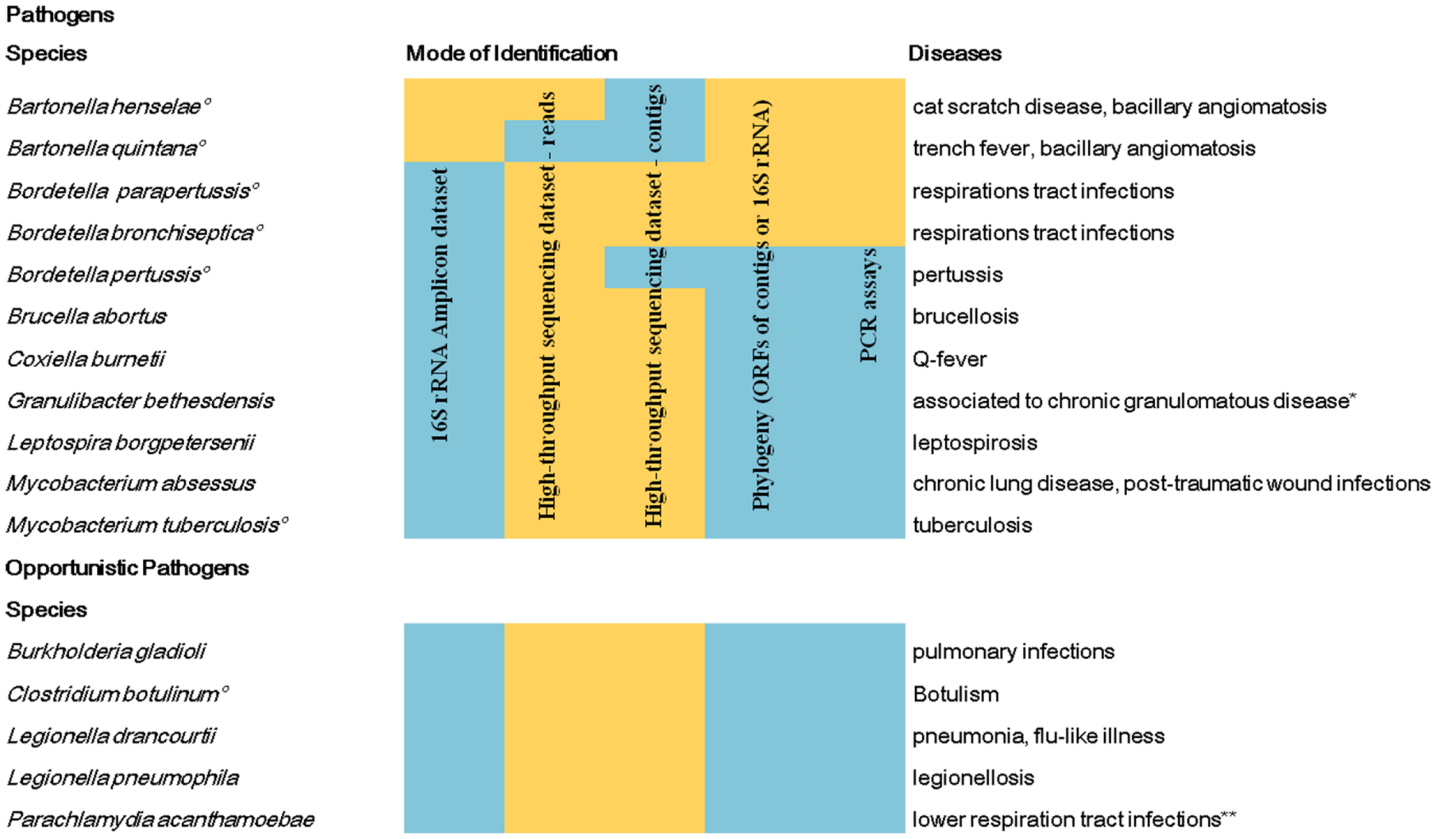
Pathogens associated with the Namur coprolite. Only human and animal pathogens detected *in silico* or by at least two tests are shown. A positive test result is marked in green and negative tests are colored in red. Pathogenic microorganisms which were previously associated to ancient human coprolites or colon contents are marked by circles. * [57] ** [58]

Some metagenomic reads were assigned to potential pathogenic bacteria (Figure 3, Tables S1 and S2B in File S1). Reads and contigs of amoeba-resistant bacteria (i.e. bacteria resisting to killing by amoeba) *Actinobacteria* spp., *Pseudomonas* spp., *Parachlamydia acanthamoebae*, *Legionella drancourtii* and *Legionella pneumophila* were found [19]. Moreover, contig annotations yielded *Burkholderia gladioli*, *Granulibacter bethesdensis*, *Leptospira borgpetersenii*, *Coxiella burnetii* and *Mycobacterium abscessus*. Contigs respectively encoding a hypothetical protein and an initiation factor 3 of *Brucella abortus* were also found, as well as reads and a contig encoding VapC46 toxin from *Mycobacterium tuberculosis* (Figure 3, Tables S1 in File S1 and S2B in File S1). Two contigs assigned to *Clostridium botulinum* encode a perosamine synthetase and a methionyl-tRNA formyltransferase. Among *Bordetella* spp. sequences found in the metagenomic dataset (Figure 3), contig reconstruction and annotation identified contigs encoding a hypothetical protein of *Bordetella parapertussis* and a putative hydrolase of *Bordetella bronchiseptica* (Tables S1, S2 in File S1). For the hydrolase, phylogenetic analyses confirmed the BLAST based annotation (Figure S3 in File S1). The cultured *P. macerans, M. luteus, S. maltophilia* and *E. cloacae* were also found in the metagenomic high-throughput sequence dataset.

In the high-throughput sequence dataset, 0.09% of reads identified bacterial 16 S rRNA genes. For further analysis, the V6 region was amplified by PCR. This 16 S rRNA gene dataset yielded 132,000 trimmed reads. The Bayesian microbial source-tracking approach was performed to compare the 16 S rRNA gene dataset associated with the Namur coprolite to those of modern stool samples, published coprolites, compost and soil as previously described [4]. The results indicated that the mixture of taxa associated to the Namur coprolite had no significant matches with any of the different sources used for comparison (Figure S4 in File S1). A total of 673 Operational Taxonomic Units (OTUs) were assigned to the 16 S rRNA gene dataset (MG-RAST accession number 4480463.3). Comparisons to the laboratory gut microbiota database including all 16 S rRNA gene sequences generated by the 454 FLX titanium platform showed that OTUs associated with the Namur coprolite did not clusterize with sequences previously amplified in the laboratory. The most abundant identified phyla were *Proteobacteria* (85.1%), *Firmicutes* (7.4%), *Actinobacteria* (2.8%) and *Chlamydia* (1.8%) (Figure 2). Furthermore, *Bartonella* species were detected with a 16 S rRNA gene sequence identity of 98.7% (Figure 3, Table S4 in File S1); phylogenetic analyses indicated that these 16 S rRNA gene amplicons were most closely related to *Bartonella henselae, Bartonella koehlerae* and *Bartonella quintana* (Table S3 in File S1and Figure S5 in File S1).

### Pathogen-specific PCR assays

Mechanical lysis of the specimen reduced PCR inhibition as measured by PCR amplification of an internal synthetic, 135-bp oligonucleotide sequence used as a PCR control. Further, microorganisms detected by microscopy or metagenomic were tentatively amplified by using species-specific PCR amplifications. Sequencing a 120-bp band generated using *Ascaris* spp.– specific primer pair [20] (Table S5 and Table S6 in File S1) yielded 98% sequence identity with *Ascaris* sp. DHS-2010a cytochrome b gene sequence, similar to those previously reported in a human remain [21] (GenBank Accession No. GU339224.1). The amplicon generated by an *Acanthamoeba* spp. – specific PCR showed a 99% sequence similarity with *Acanthamoeba castellanii* (GenBank Accession No. JF437606.1) [22]. *Bartonella* species, detected in the 16 S rRNA gene rDNA dataset, were additionally amplified by PCR. The amplicon of the ribosomal RNA operon *rrsC* yielded 95% of sequence identity with *B. henselae* and 94% with *B. quintana* (GenBank Accession No. BX897699.1 and BX897700.1), respectively. *Bordetella* species, detected in the high-throughput sequencing dataset, were also amplified in the PCR assays (Figure 3). A 189-bp long fragment of the RNA polymerase C gene was generated, exhibiting 99% of sequence similarity with *B. bronchiseptica* (GenBank Accession No. HE965806.1, BX640437.1, HE965807.1), *B. parapertussis* (GenBank Accession No. HE965803.1, BX640423.1) and *Achromobacter xylosoxidans* (GenBank Accession No. CP002287.1). The obtained amplicon differed in one position (T → C transition) from the *B*. *parapertussis* and *B. bronchiseptica* reference strains. In two positions, the amplicon differed from *B. bronchiseptica* MO149 strain (T → C transition) as from the reference sequence of *A. xylosoxidans* (T → C transition and G → C mutation) (Figure S6 in File S1) respectively.

### Synopsis of identified microorganisms

The identified microorganisms were categorized into two groups. The first group comprises microorganisms for which the identification was confirmed by at least two independent methods among microscopy, culture, metagenomic and the PCR assay. This group includes *Ascaris* spp. and *Acanthamoeba* spp. identified by microscopy and PCR assay; *P. macerans, M. luteus*, *S. maltophilia* and *E. cloacae* found by culture and metagenomic; and *Bartonella* spp. – related to *B. henselae* and *B. quintana* – and *Bordetella* spp. found in metagenomic or 16 S rRNA gene amplicon datasets and the PCR assay (Figure 3). The second group comprises of microorganisms identified only *in silico* by contig annotations (Figure 3). This group includes *B. abortus*, *C. botulinum*, *C. burnetii* ¸ *G. bethesdensis*, *M. tuberculosis*, *M. abscessus*, *B. gladioli*, *L. drancourtii*, *L. pneumophila*, *L. borgpetersenii*, *P. acanthamoebae*, *B. finegoldii*, *B. vulgatus*, *B. coprocola* and *B. coprosuis* (Figure S2 in File S1).

## Discussion

Paleomicrobiological investigations of a total of fourteen human coprolites and one colon sample have been reported from six different American archeological sites [2], [4], [5], [7], [10]. As for Europe, only two colon content specimens from two different sites, respectively dated to 3,350–3,100 BC and 1918 AD and no coprolite have been analyzed [8], [9]. In the present study, a coprolite collected from a medieval site in Belgium was investigated using a polyphasic approach.

This coprolite was recovered from a sealed barrel which was still intact at the time of its discovery, and only the internal portion of the coprolite was investigated. Current recommendations for paleomicrobiology and paleoparasitology studies were strictly enforced in order to minimize in-laboratory contaminations [1], [23]–[26]. No positive control was used and negative controls incorporated during all the experimentations remained negative. Nevertheless, the data herein reported – the presence of amoebal cysts, plant fibers, seeds, pollens and mold remains – indicate that the coprolite obviously contained environmental flora as previously reported in other investigations [4], [5], [10], [27]. Accordingly, source tracking of the 16 S rRNA gene amplicon dataset yielded no hits, as previously also observed for two pre-Columbian American coprolites [4]. More coprolites need to be investigated before source tracking can be informative. Furthermore, the observation of pig- and human-infecting *Trichuris* spp. eggs in the coprolite indicates a possible common use of the barrel for animal and human feces. This point was supported by the results obtained in the metagenomic datasets, with bacterial sequences assigned to species found in modern gut microbiota of humans and pigs. Nevertheless, parts of the Namur coprolite correspond to human gut microbiota. Gut bacteria phyla – *Alpha*-, *Beta*- and *Gammaproteobacteria* and *Bacteroidetes* – were found herein as described for ancient coprolites and colon contents from American and European archeological sites [4], [5], [9], [10]. Furthermore, *Corynebacterium*, *Enterobacter* and *Prevotella* are common inhabitants of the modern human digestive tract, further detected in the Namur coprolite [4], [5], [9], [10], [28]. Moreover, intestinal human parasites including *Trichuris* and *Ascaris* were also identified. The sequence obtained for the intestinal parasite *Ascaris* spp. supports the human origin of the Namur coprolite. Indeed, the same *Ascaris* spp. sequences were previously amplified from the remains of a human pelvic bone from a medieval Korean tomb [21].

On this basis, the Namur coprolite was further used as a source to detect past pathogens. Modern stool specimens diagnose systemic infections in humans and primates including malaria, rickettsiosis and tuberculosis [29]–[31]. Likewise, two pathogens *Haemophilus parainfluenzae* and *Clostridium botulinum* were already found in ancient fecal material [2], [7] and *Neisseria, Yersinia, Shigella* and *Mycobacterium* sequences were identified in WGS datasets of pre-Columbian American coprolites [5]. To go further, it was herein attempted to ascertain the presence of potential pathogenic agents that might be associated to the analyzed specimen, *in silico* or by molecular tests. Bacterial pathogens belonging to *Bartonella* and *Bordetella* were detected here for the first time in a coprolite sample. *Bartonella* species are responsible for zoonoses [32] and *Bordetella* species cause respiratory tract infections [33].

## Conclusion

Using a polyphasic approach including culture-dependent and culture-independent techniques, several microorganisms were consistently identified in one coprolite dating back to the European Middle Ages. These microorganisms included both common members of the gut microbiota and systemic pathogens. Coprolites are a source of knowledge regarding both microbiota and pathogens circulating in ancient populations and could be investigated using techniques routinely used for the modern diagnosis in clinical microbiology.

## Materials and Methods

### Ethic statement

According to the French legislation, no permits are required for the investigation of coprolite in general and for this study in particular.

### Contamination prevention

After excavation, the coprolite was stored in a sterile forensic specimen bag. In 2006, the coprolite was sent to our laboratory, where it was handled only in a positive pressure room with isolated ventilation under strict aseptic conditions. Workbenches were stringently disinfected using absolute ethanol and UV-irradiation for at least 30 min. Non-disposable instruments were autoclaved. Reagents and chemicals were from new stocks aliquoted into sterile, single-use tubes and immediately discarded after use. The external portion of the coprolite was aseptically removed; only the internal portion of the coprolite was used. DNA -extraction, PCR and post-PCR experiments were performed in separate rooms in isolated work areas. Positive controls were strictly avoided. Negative controls for DNA -extraction and PCR were used at a 1∶4 control: specimen ratio [1], [23]–[26].

### Microscopy

A 350-mg sample taken from the interior portion of the coprolite specimen was rehydrated for three days in 5 mL of phosphate buffered saline (PAS;Biotechnologie Appliquée, Taden, France) *prior* to observation under a microscope. Congo-red staining was applied to stain the cellulosic material of the coprolite. Then, 500 mg samples were taken from the interior of the coprolite specimen and rehydrated for two days in 5 mL of PAS. The diluted samples were fixed with absolute methanol on a glass slide, coated with 1 mg/mL Congo-red solution (Microm, Francheville, France) and incubated for 1 h at room temperature. The slide was carefully rinsed with 1 M sodium hydroxide (Aldrich-Chemie GmbH, Steinheim, Germany) and air-dried. Microscopic observations were performed using a Leica DM2500 microscope (Leica Microsystems, Nanterre, France) at 40× and 100× magnifications. Pictures were taken using a Nikon digital sight DS-U1 camera with Lucia G software (Nikon Instruments, Champigny sur Marne, France). Measurements were collected using the ImageJ software (http://rsbweb.nih.gov/ij/). Further, 500 mg of the coprolite were rehydrated into 1 mL PBS (Biotechnologie Appliquée), fixed with absolute ethanol on glass slides and stained using acridine orange solution as previously described [10].

### DNA recovery from the coprolite

Two independent extraction protocols were used to obtain ancient DNA of the widest possible length range and to remove pigments, inhibitors of molecular detection methods. One gram of the coprolite was solubilized overnight at 4°C in 1 mL TE buffer (ethylenediamine tetraacetic acid; buffered solution, Tris HCl 10 mM, EDTA 1 mM, pH 8). A 500-µL aliquot of the solution was used for total DNA extraction as previously described [34], except that incubations into TE and digestion buffers were shortened to 1 day. TE buffer without coprolite was used as a negative control. For the DNA extraction using the PowerSoilR DNA Isolation Kit (MoBio Laboratories, Inc., Carlsbad, USA) [5], 500 µL of solubilized coprolite specimen were also used. Incubation was extended to 24 h/56°C in PowerSoil^R^ bead tubes containing sodium dodecyl sulfate (SDS) and digestion buffer C1, under contentiously rotation followed by shaking in a Bio 101 FastPrep instrument (Qbiogene) at level 6.5 (full speed) for 95 s. DNA extraction was performed according to the manufacturer's instructions. Extraction batches contained also negative controls composed of PowerSoil^R^ bead tubes without coprolite. Total DNA extracts from both protocols above were pooled together.

### Culture

The coprolite was inoculated into broth in vertical tubes producing a gradient of oxygen, to recover aerobic, microaerophilic and anaerobes bacteria which may have survived inside the coprolite. Schaedler (Neogen, Lansingen, Michigan) and R2A (Neogen) broths with an additive of 1.5% of agarose were used as culture media. Sterile screw capped tubes (BIO-RAD, Marnes-la-Coquette, France) were filled with 15 mL of growth media and boiled for 30 min and then incubated at 47°C for 10 min. A 1-g inner portion of the coprolite was removed under anaerobic conditions and solubilized in 2 mL of sterile PBS (Biotechnologie Appliquée). Then, 200 µL of this suspension were injected into tubes containing Schaedler agar tubes under strict anaerobic atmosphere. In parallel, 1 mL of this suspension was incubated at 70°C for 20 min and then injected under strict anaerobiosis into tubes containing R2A broth. Tubes were incubated at 30°C for daily inspection. When visible growth was observed, the tubes were sliced with a sterile glass cutter under strict anaerobiosis and subcultured on Schaedler or R2A agar plates under various atmospheres. The appropriated atmospheres were created in sterile incubation bags using gas generating pouch systems (BD Gas PakTM EZ, Maryland, USA). During the entire procedure, two negative controls (broth with and without PBS were carried out. Additionally, BD BACTEC, Lytic/10 Anaerobic and Aerobic bottles enriched with 7 mL of defibrinated sheep blood (bioMérieux, Marcy l'Etoile, France) were used for culturing. 1-g of the inner portion of the coprolite was solubilized into 1 mL of PBS (Biotechnologie Appliquée) and 300 µL of such suspension were injected into the culture bottle. After 2-day incubation at 37°C, 100 µL of the anaerobic and aerobic culture liquid were serially diluted (D1:D10^−10^) and 10 µL of each dilution were plated onto COS culture plates (bioMérieux) and incubated at 37°C under strict anaerobe, microaerophile and aerobe atmosphere created by the use of gas generating pouch systems. Culture bottles with 300 µL of sterile PBS were run in parallel as negative controls. Likewise, COS plates (bioMérieux) inoculated with 10 µL culture liquid form the negative bottle culture and a COS plates (bioMérieux) that were opened under the Biosafety cabinet level 2 during the whole time of manipulation, were used as negative controls during subculture. Colonies were identified by MALDI-TOF mass spectrometry. Briefly, colonies were directly spotted on the mass-spectrometer plate and overlaid by appropriate matrix, before introduction into a Microflex mass spectrometer (Bruker Daltonics, Wissembourg, France). The spectrum of 2,000 – 20,000 – dalton peaks was compared to those in the database, which comprised of 3,768 reference bacterial spectra, in order to achieve identification [15]. When MALDI-TOF identification failed, colonies were identified by 16 S rRNA gene sequence analysis as previously described [16].

### High-throughput pyrosequencing of the 16 S rRNA gene V6 region

The V6 region was amplified using 454-adapter primers [35]. PCR was performed in a final volume of 50 µL containing 1×PCR buffer, 2 µL of 25 mM MgCl_2_, 200 µM of each d’NTP, 1 µL of 10 pM of each primer (10 pM), 31.15 µL ddH2O, 1 unit of HotStar Taq Polymerase (Invitrogen, Villebon sur Yvette, France) and 57–112 ng of DNA-extract. The amplification was performed by incubating at 95°C for 15 min, followed by 31 cycles of denaturation at 95°C for 45 sec, annealing at 58°C for 45 sec, elongation at 72°C for 90 sec, followed by an final elongation at 72°C for 10 min in an ABI Thermocycler (Applied Biosystems Gene Amp PCR System 2700, Villbon sur Yvette, France). PCR products were purified using Ampure beads (AgentcourtR AMPureR XP, Beckman Coulter, USA) and checked using a BioAnalyzer (Agilent 2,100 Bioanalyzer, Agilent Technology, Lithuania) LabChip with a DNA chip 7,500 at 548 bp (Agilent DNA 7,500 Reagent). Quantification of PCR products was performed using a Tecan GENios fluorometer was perfomed using a Quant-iTTM PicoGreenR ds DNA Assay Kit (Invitrogen) following the Amplicon Library Preparation Method in a manual provided by Roche (Roche, Mannheim, Germany). The concentration of the library was 27.7 ng/µL, corresponding to 8.84 E×10 molecules/µL. Clone amplification was performed using a GS Titanium LV emPCR Kit (Lib-A) v2 using only DNA Capture Beads A (Roche). Sequencing was performed with a GS FLX Titanium XLR70 Sequencing Kit (Roche).

### OTU-based analysis

The 16 S rRNA gene pyrosequencing data were processed using Mothur package 1.5 [36]. No ambiguous bases ‘N’ and only one mismatch were allowed in the read and primer sequences. The quality read trimming used a moving window of 50 bp and required that the average quality score over the region did not drop below 35. The trimmed reads were dereplicated and aligned using the Sylva bacteria reference alignment provided by the Mothur (http://www.mothur.org/). The multiple sequence alignment was filtered using a minimum read length of 200 bp. In addition, a pre-clustering step [37] was performed before chimera detection using the Uchime tool in Mothur. A distance matrix was built based on multiple sequence alignment, and OTUs clustering was performed at a 97% sequence identity. The taxonomic classification from phylum to genus level of each representative OTU sequence was performed using the RDP classifier tool and the RDP training set 9-database (http://www.mothur.org/). The relative abundance of reads per phyla was deduced from this classification. To exclude laboratory contaminations, we built an in-house gut microbiota database. This gut microbiota database contained 5,500,000 16 S rRNA gene sequences using all of the data generated by the 454FLX titanium platform of the URMITE laboratory. The database includes data from obese (SRX118214), Senegalese (SRX118212, SRX118213), HIV (SRX209782), anorexic (SRX209240), post- antibiotic treatment (SRX189054, SRX189053) and drug resistance tuberculosis (SRX204218) stool specimens. The OTUs identified in the Namur coprolite were clustered at 97% sequence identity with sequences in our gut microbiota database. The OTUs and corresponding sequences that did not match with any sequence of the database were evaluated.

### Phylotype-based analysis

To detect potential pathogens in the 16 S rRNA gene pyrosequencing data, we used an alternative method that consisted of binning reads according to their taxonomic classification using BLAST searches against the Ribosomal Database Project (RDP) database. First, the OTU-based quality read trimming filter was applied. However, no multiple sequence alignment and no distance matrix were performed to increase read length and therefore, improve the ability of assignment at the species level. Two databases were created using selected criteria from the Hierarchy Browser of the RDP 16 S rRNA database, release 10 (http://rdp.cme.msu.edu/). A “Type” database was built with sequences labeled “Type strains”, “Isolates” and size “length >1,200 bp” with “good quality”. A “Non-type” database was built using “Non-type strains”, “Isolates”, size “length >1,200 bp” and “good quality”. The two databases were formatted using Taxcollector [38]. The species level was defined with a minimum sequence identity of 98.7% [39] with the best BLAST hit from the “Type” database. The multiple best BLAST hit cases were checked for the most representative species (>50% of the multiple best BLAST hits). A second BLAST round was performed from the remaining reads with the same cutoffs but using the “Non-type” database. A total of 121 reads were assigned to the genus *Bartonella*, including 53 reads that were significantly assigned to *B. quintana* and 47 reads to *B. henselae*. For some 16 S rDNA amplicons phylogenetic trees were constructed. Multiple sequence alignment was performed using MUSCLE [40] and curated by Gblocks [41]. The phylogenetic tree was built using the PhyML algorithm [42] with a bootstrap of 100 and the nucleotide substitution model HKY85 [43]. These tasks were all performed using the pipeline www.phylogenie.fr [44]. The phylogenetic trees were visualized using trees DrawTree [45].

### Source-tracking analysis

To determine the potential sources of bacteria contained in the coprolite sample (defined as sink) we used the SourceTracker software package [46] as previously reported [47]. The Source Tracker sofware uses a Bayesian model that estimate the different source proportion found in a community sample (sink). The different sources examined (16 S rRNA datasets) correspond to 88 soil samples [48], 602 multiple human body sites (skin, nasal, tongues, urine) including 45 U.S. gut samples [49], 20 infant guts [50], 3 Polynesian guts from our laboratory, 60 mammal guts [51], eight different coprolites [4] and one compost [52]. If the community tested corresponds to a mixture of taxa that do not match with any of the source environments used, that portion of the community is classified as “unknown”. The 16 S rRNA reads were processed for all source and sink samples using the quantitative insights into microbial ecology 1.7.0 release (QIIME) [53]. High-quality read sequences (quality score >30, exact match to primer, and containing no ambiguous characters) were trimmed from the initial dataset. When compared datasets from non-overlapping amplicons the taxonomy binning with the closed-reference OTU picking strategy and using the Greengene gg_otus-12_10 release were performed. Then, all the taxonomy tables were converted and merged, for all the mapping files of the various source/sink datasets for SourceTracker analysis with the defaults parameters (iterations for Gibbs sampling  = 100, rarefaction depth  = 1000, alpha1 = 1e^−3^, alpha2 = 1e^−1^). The modification of the Source tracker parameters did not change the results obtained for the analysis of the Namur coprolite. To control the analysis a soil sample [48] and a previously investigated coprolite ZA04 were used as positive controls [4] (Figure S4 in File S1). The coprolite specimen matched to the Sources of primate/mammalian gut and Burkina Faso children gut.

### High-throughput metagenomics

The shotgun strategy was chosen for high-throughput pyrosequencing on a 454 Life Sciences Genome sequencer FLX instrument using titanium chemistry (Genome Sequencer RLX, Roche). Sequencing was performed using eight regions of the PicoTiterPlate. The concentration of extracted DNA was measured with the QuAnt_IT Picogreen Kit (Invitrogen) on a Tecan Fluorometer (GENios) at a concentration of 28.2 ng/µL. A total of 500 ng of DNA was nebulized. The library was constructed according to the 454-titanium shotgun protocol and the manufacturer's instructions. DNA fragmentation was visualized using the BioAnalyzer 2,100 on a LabChip with high sensitivity and an optimal size of 872 bp. The DNA stock was measured on a TBS fluorometer at 8.776 E+08 molecules/µL. The library was clonally amplified with 3cpb in 3 emPCR reactions using the GS Titanium SV emPCR Kit (Lib-L) version 2.The titration yield was 12.31%. In total 340,000 beads per project and per region were loaded onto the GS Titanium PicoTiterPlate Kit 70×75 which corresponded for this project with 383 µL of the clonal amplification and sequenced with the GS Titanium Sequencing Kit XLR70. The run was performed overnight, and then analyzed using the cluster. Using the Camera 2 [54] QC-Filter and the 454 duplicate clustering tool, reads that were low quality (average score <19) or that were <60 bp and identical duplicates (default sequence identity  = 0.96) artificially produced by titanium technology, were deleted. Reads were blasted against the NCBI non-redundant protein database using a translated nucleotide query (BLASTX). The best BLAST-hits with ≥50% identity, ≥50 score and E-values <1e^−05^ [17], [18] were retained. Reads were assembled reads into contigs using a GS De Novo Assembler (Roche) with the following parameters: minimum overlap length of 35 bp, minimum identity of 98%. ORF searching was performed using Prodigal [55], and ORFs were blasted against the NCBI non-redundant database (BLASTP, E<1e^−05^). When possible, phylogenetic trees of the ORFs of contigs, encoding proteins that were associated to potential bacterial pathogens, were built. Regions that were homologous to the translated ORFs were searched using BLASTP against the non-redundant NCBI database. For multiple alignments of the sequences MUSCLE [40] was used and then curated by Gblocks [41]. The phylogenetic tree was built using the PhyML algorithm [42] with a bootstrap of 100 and the protein substitution model WAG. These tasks were all performed using the pipeline www.phylogenie.fr [44]. For the visualization of the phylogenetic trees DrawTree [45] was used. Principal coordinates analysis was performed using the correlated tool on the MG-RAST [56] metagenomic analysis server, to evaluate similarity between coprolite, soil and modern feces samples at the metabolic taxonomic level. Metabolic classifications were generated by a BLAST search against the SEED database using an E-value <1e -05, a minimum identity cutoff of 80% and a minimum alignment length cutoff of 20 bp. The data were normalized to values between 0 and 1, distances were calculated using the Bray-Curtis method. The results of the analysis are represented in the Figure S7 in File S1.

## Supporting Information

File S1 **Supporting information. Note S1.** **Specific PCR-amplifications and Sanger-sequencing, quantitative real time PCR.** **Table S1.** **Typical gastro-intestinal, environmental and pathogenic bacteria assigned to the coprolite metagenome^#^.** ^#^Metagenomic reads were blasted against the NCBI protein database using a translated nucleotide query. Underrepresented taxonomic genera are not shown. **Table S2.** **Number and size of contigs that were assigned to (A)** ***Bacteroides*** **spp. and (B) to bacterial pathogens associated to the coprolite sample^#^.** ^#^The contig identifier, its length (bp) and the annotation according to the best BLAST hit (BLASTX *versus* the non-redundant NCBI database, E-value<1e^−05^) are summarized. The E-value, the hit accession identifier and the percent of identity are also provided. Contigs of °human and *pig gut microbiota *Bacteroides* species. **Table S3.** **Cultured microorganisms^#^.** ^#^ Reported are the culture conditions, the cultured microorganisms and the mode leading to species identification. Reported is also if the cultured bacteria were found in the high-throughput pyrosequencing dataset. ° When the identification was based on BLAST annotation of the amplified 16 S rRNA gene region additional phylogenetic trees were constructed (Figures S2). **Table S4**. **Bacterial pathogens identified from the amplified 16 S rRNA V6 region^#^. ^#^** The species level was defined with a minimum sequence identity of 98.7% using BLAST similarity searches against RDP databases. **Table S5. Primers used to amplify DNA from intestinal parasites, bacterial pathogens and amoebae. Table S6.** **The quantitative real-time PCR systems that were tested. Figure S1. Working overview of the polyphasic approach used to analyze the Namur coprolite. Figure S2.** **Phylogenetic of 16 S rRNA gene sequences generated form cultivated bacteria species.** The tree was constructed using the PhyML algorithm with a bootstrap of 100. The bootstrap support is reported for each branch. Phylogenetic tree of 16 S rDNA amplicons closely related to (A) *Bacillus horti*, (B) *Paenibacillus* spp., (C) *Rhodanobacter* spp. and (D) *Clostridium magnum*. **Figure S3. Phylogenetic tree of a hydrolase.** A phylogenetic tree was generated from the translated open reading frame of a contig encoding a hydrolase close to *Bordetella* species. The tree was constructed using the PhyML algorithm with a bootstrap of 100. The bootstrap support is reported for each branch. **Figure S4. Bayesian source-tracking results.** (A) The mixture of taxa associated to the 16 S rDNA gene amplicon dataset of the coprolite specimen was compared to known dataset of various environments. To control the workflow used to perform the analyses, two known samples (B) one coprolite previously investigated [9] and (C) a soil sample [10] were positively tested. **Figure S5. Phylogenetic tree of 16 S rDNA amplicons matching to** ***Bartonella sp.*** The tree was constructed using the PhyML algorithm with a bootstrap of 100. The bootstraps are reported for each branch. Phylogenetic tree of 16 S rDNA amplicons closely related to (A) *B. henselae*, *B. koehlerae* and (B) *B. quintana*. **Figure S6. Alignment and of the amplicon matching to** ***Bordetella*** **and** ***Achromobacter*** **.** The sequence alignment was performed using CLUSTALW multiple alignment tool [11]. **Figure S7.** **Metabolic comparison of modern metagenomes to the coprolite metagenome.** The Principal coordinates analysis was based on read classification according to BLASTX searches against the SEED Database. For each metagenome included the MG-RAST accession number is given. Compared metagenomes are from soil (yellow cluster), healthy mammalian and human feces (blue cluster); and the coprolite (red). The coprolite metagenome does not group with either the modern gut or soil microbiota.
(DOCX)
