# Supplementary material for: Polyphasic Analysis of a Middle Ages Coprolite Microbiota, Belgium
Source: PLoS One. 2014 Feb 28;9(2):e88376. doi: 10.1371/journal.pone.0088376 (PMC3938422; doi:10.1371/journal.pone.0088376)
Supplement: File S1 — Supporting information. Note S1. Specific PCR-amplifications and Sanger-sequencing, quantitative real time PCR. Table S1. Typical gastro-intestinal, environmental and pathogenic bacteria assigned to the coprolite metagenome#. #Metagenomic reads were blasted against the NCBI protein database using a translated nucleotide query. Underrepresented taxonomic genera are not shown. Table S2. Number and size of contigs that were assigned to (A) Bacteroides spp. and (B) to bacterial pathogens associated to the coprolite sample#. #The contig identifier, its length (bp) and the annotation according to the best BLAST hit (BLASTX versus the non-redundant NCBI database, E-value<1e−05) are summarized. The E-value, the hit accession identifier and the percent of identity are also provided. Contigs of °human and *pig gut microbiota Bacteroides species. Table S3. Cultured microorganisms#. # Reported are the culture conditions, the cultured microorganisms and the mode leading to species identification. Reported is also if the cultured bacteria were found in the high-throughput pyrosequencing dataset. ° When the identification was based on BLAST annotation of the amplified 16 S rRNA gene region additional phylogenetic trees were constructed (Figures S2). Table S4. Bacterial pathogens identified from the amplified 16 S rRNA V6 region#. # The species level was defined with a minimum sequence identity of 98.7% using BLAST similarity searches against RDP databases. Table S5. Primers used to amplify DNA from intestinal parasites, bacterial pathogens and amoebae. Table S6. The quantitative real-time PCR systems that were tested. Figure S1. Working overview of the polyphasic approach used to analyze the Namur coprolite. Figure S2. Phylogenetic of 16 S rRNA gene sequences generated form cultivated bacteria species. The tree was constructed using the PhyML algorithm with a bootstrap of 100. The bootstrap support is reported for each branch. Phylogenetic tree of 16 S rDNA amplicons closely relat [file pone.0088376.s001.docx]

**SUPPORTING INFORMATION FILE**

**Polyphasic analysis of a Middle Age coprolite microbiota, Belgium**

Sandra Appelt^1^, Fabrice Armougom^1^, Matthieu Le Bailly^2^, Catherine Robert^1^, Michel Drancourt^1*^

^1^ Aix Marseille Université, URMITE, UM63, CNRS 7278, IRD 198, Inserm 1095, 13005 Marseille, France.

^2^ Franche-Comté University, CNRS UMR 6249 Chrono-Environment, 25 030 Besançon, France.

*Corresponding author:

Michel Drancourt, URMITE, UM63, Faculté de Médecine, 27 Bd Jean Moulin, 13385 Marseille cedex 5, France, Phone (33) 491 38 55 17, Fax (33) 491 38 77 72.

Email: Michel.Drancourt@medicine.univ-mrs.fr

**SUPPLEMENTARY INFORMATION FILE CONTENTS :**

**SUPPLEMENTARY NOTE**

**SUPPLEMENTARY FIGURES AND TABLES**

**Supplementary Tables S1-S6**

**Supplementary Figures S1-S7**

**SUPPLEMENTARY REFERENCES**

**Supplementary note**

**SUPPLEMENTARY MATERIAL SECTION. S**pecific PCR-amplifications and Sanger-sequencing, quantitative real time PCR.

**Specific PCR-amplifications and Sanger-sequencing**

Suicide PCR –amplification [[1](#_ENREF_1)] was performed to reinforce the high-throughput pyrosequencing results and perform target-orientated searches for *Ascaris* sp., amoebae and viridae. For the molecular detection of *Ascaris* sp., a 145-bp region of the cytochrome b gene was amplified [[2](#_ENREF_2)]. To detect *Acanthamoeba* spp., we targeted the *Acanthamoeba* spp. 18S rRNA gene [[3](#_ENREF_3)]. In addition, seven primer pairs specific for the RNA-polymerase gene C of *Bordetella* sp., 16S rRNA gene of *Mycobacterium kumamotonense,* *Rickettsia* sp.*,* *Ehrlichia* sp.*, Brucella* sp., and *Bartonella* sp. were designed. The primers are summarized in **Supplementary Table S6**. Additionally an internal in-laboratory control was included, to check for PCR inhibitors in the DNA extract. PCR was performed in a 50-µL final volume containing 1x PCR buffer, 2 µL of 25 mM MgCl_2_, 200 µM of each d’NTP, 1 μL of 10 pM of each primer, 31.15 µL of ddH_2_O, 1 unit of HotStar Taq Polymerase (Invitrogen, Villebon sur Yvette, France) and 57-112 ng of DNA-extract. The steps of the PCR were an initial incubation at 95°C for 15 min; 36-40 cycles of denaturation for 95°C for 1 min, annealing for 45 sec at the corresponding primer annealing temperatures, and elongation at 72°C for 90 sec and a final elongation 72°C for 10 min; all of these steps were performed in a Gene Amp PCR System 2700 ABI Thermocycler (Applied Biosystems, Villbon sur Yvette, France). The PCR products were analyzed using a 2 % agarose gel (UltraPureTM agarose, Invitrogen, Villbon sur Yvette, France) and purified using the QIAquick PCR Purification Kit (QIAGEN, Courtaboeuf, France). For the F6-R6 and F8-R8 primer pairs, the PCR products were cloned into the pEGM-T-Easy Vector system I (Promega, Carbonnieres, France) and transferred *via* heat shock into competent JM109 *Escherichia coli* cells according to the manufacturer’s instructions. White colonies were screened on LB-Amp-XGal-IPTG agar plates using the following M13 PCR primers M13F 5’-GTAAAACGACGGCCAG-3’ and M13R 5’-CAGGAAACAGCTATGAC-3’. All of the PCR and cloning products were sequenced in a final volume of 20 of µL (1 x sequencing buffer, 3.2 pM forward or reverse primer, 4 µL of BigDye Terminator V1.1 mix (Applied Biosystems), 7.4 µL of ddH_2_O and 4 µL of PCR product) after purification using Sephadex Gel Filtration in the ABI PR ISM 3130xl genetic sequencer (Applied Biosystems, Villbon sur Yvette, France). The sequences were assembled using the ChromasPro software and compared with reference sequences of the GenBank database using NCBI BLAST searches.

**Quantitative real time PCR amplifications**

Quantitative real-time PCR (qPCR) assays targeting various genomic regions of twenty-two pathogens were performed to examine for their presence in the coprolite specimen. All of the targeted microorganisms and genes, including the primer pairs and probes, are listed in Supplementary Table S8. Serial ten-fold dilutions (from 10 ^-1^ to 10^-3^) of the DNA extract derived from the coprolite specimen were performed to dilute potential molecular detection inhibitors in the brown DNA supernatant. The primers and probes were used at final concentrations of 500 nM and 62.5 nM, respectively. For the qPCR mix, we used a commercial MasterMix of Eurogentec (Eurogentec, Angers, France) according to the manufacturer’s instructions. The reactions were performed using a C1000TM Thermal cycler (CFX96TM Real-Time System, BIORAD, Marnes-la-Coquette, France). Negative controls consisting of the reaction mix without DNA were added in a ratio of 1:3. To avoid contamination, the experiments were performed in working areas in which these systems had never been used. Overall, 30 different qPCR diagnostic systems were tested using various ten-fold dilutions of the coprolite DNA extract. Altogether, 30 different qPCR diagnostic systems were tested using various ten-fold dilutions of the coprolite DNA extract. All of the tested systems and all of the negative controls remained negative.

**Table S1.** **Typical gastro-intestinal, environmental and pathogenic bacteria assigned to the coprolite metagenome^#^.**

|  | Gastro-intestinal Bacteria | | |  | | Environmental Bacteria | | |  | | Bacterial Pathogens | | | | | |  | | |  | | | | | Opportunistic Pathogens      Pathogens | | | | |
| --- | --- | --- | --- | --- | --- | --- | --- | --- | --- | --- | --- | --- | --- | --- | --- | --- | --- | --- | --- | --- | --- | --- | --- | --- | --- | --- | --- | --- | --- |
| Genus (Reads) | | **Species** | **Reads** | | **Genus (Reads)** | | **Species** | **Reads** | **Genus (Reads)** | | | | **Species** | | | | | **Reads** | | | | **Genus (Reads)** | | | | **Species** | | **Reads** | |
| Aeromonas (7) | | *A. salmonicida* | 4 | | Acidovorax (76) | | *A. avenae* | 16 | Bartonella (9) | | | | *B. henselae* | | | | | 1 | | | | Achromobacter (114) | | | | *A. xylosoxidans* | | 80 | |
|  | | *A. veronii* | 3 | |  | | *A. citrulli* | 7 |  | | | | *B. rochalimae* | | | | | 3 | | | |  | | | | *A. piechaudii* | | 34 | |
| Bacteroides (82) | | *B. cellulosilyticus* | 6 | |  | | *A. delafieldii* | 28 |  | | | | *Bartonella* sp. | | | | | 3 | | | | Actinobacter (20) | | | | *A. baumannii* | | 4 | |
|  | | *B. fragilis* | 5 | | Agrobacterium (199) | | *A. radiobacter* | 93 | Bordetella (153) | | | | *B. avium* | | | | | 15 | | | | Aeromonas (9) | | | | *A. veronii* | | 3 | |
|  | | *B. intestinalis* | 5 | |  | | *A. tumefaciens* | 41 |  | | | | *B. bronchiseptica* | | | | | 37 | | | |  | | | | *A. hydrphila* | | 2 | |
|  | | *B. plebeius* | 5 | |  | | *A. vitis* | 38 |  | | | | *B. parapertussis* | | | | | 16 | | | | Bacillus (112) | | | | *B. cereus* | | 14 | |
|  | | *Bacteroides* sp. | 30 | | Beutenbergia (10) | | *B. cavernae* | 10 |  | | | | *B. pertussis* | | | | | 14 | | | |  | | | | *B. thuringiensis* | | 7 | |
| Clostridium (45) | | *C. botulinum* | 6 | | Burkholderia (832) | | *B. cenocepacia* | 36 | Brucella (164) | | | | *B. abortus* | | | | | 9 | | | | Burkholderia (832) | | | | *B. gladioli* | | 26 | |
|  | | *C. difficile* | 3 | |  | | *B. gladioli* | 26 |  | | | | *B. melitensis* | | | | | 10 | | | |  | | | | *B. mallei* | | 4 | |
|  | | *C. leptum* | 4 | |  | | *B. multivorans* | 34 |  | | | | *B. neotomae* | | | | | 2 | | | |  | | | | *B. pseudomallei* | | 41 | |
| Collinsella (5) | | *C. aerofaciens* | 4 | |  | | *B. pseudomallei* | 41 |  | | | | *B. ovis* | | | | | 4 | | | | Clostridium (45) | | | | *C. botulinum* | | 6 | |
| Corynebacterium (25) | | *C. aurimucosum* | 10 | |  | | *B. phytofirmans* | 34 |  | | | | *B. suis* | | | | | 5 | | | | Comamonas (20) | | | | *C. testosteroni* | | 19 | |
| Eikenella (6) | | *E. corrodens* | 3 | |  | | *B. ubonensis* | 35 | Chlamydia (7) | | | | *C. pneumoniae* | | | | | 1 | | | | Delftia (23) | | | | *D. acidovorans* | | 22 | |
|  | | *E. billigiae* | 3 | |  | | *B. graminis* | 46 |  | | | | *C. trachomatis* | | | | | 3 | | | | Escherichia (27) | | | | *E. coli* | | 21 | |
| Enterobacter (15) | | *E. cancerogenus* | 1 | |  | | *B. phymatum* | 59 |  | | | | *C. psittaci* | | | | | 2 | | | | Legionella (36) | | | | *L. pneumophila* | | 18 | |
|  | | *E. cloacae* | 9 | |  | | *B. xenovorans* | 72 | Coxiella (13) | | | | *C. burnetii* | | | | | 13 | | | |  | | | | *L. drancourtii* | | 7 | |
|  | | *Enterobacter* sp. | 5 | | Chromobacterium (25) | | *C. violaceum* | 25 | Granulibacter (45) | | | | *G. bethesdensis* | | | | | 45 | | | | Parachlamydia (31) | | | | *P. acanthamoebae* | | 31 | |
| Escherichia (27) | | *E. albertii* | 3 | | Erythorbacter (170) | | *E. litoralis* | 63 | Klebsiella (10) | | | | *K. pneumoniae* | | | | | 4 | | | | Pseudomonas (292) | | | | *P. aeruginosa* | | 47 | |
|  | | *E. coli* | 21 | |  | | *Erythorbacter* sp. | 107 | Leptospira (19) | | | | *L. borgpetersenii* | | | | | 6 | | | |  | | | | *P. putida* | | 29 | |
|  | | *E. fergusonii* | 3 | | Gemmata (59) | | *G. obscuriglobus* | 57 | Mycobacterium (113) | | | | *M. intracellulare* | | | | | 9 | | | |  | | | | *P. stutzeri* | | 37 | |
| Eubacterium (6) | | *E. limosum* | 4 | | Gemmatimonas (5,370) | | *G. aurantiaca* | 5,370 |  | | | | *M. tuberculosis* | | | | | 9 | | | | Rhodococcus (65) | | | | *R. equi* | | 21 | |
| Flavobacterium (58) | | *F. johnsoniae* | 28 | | Laribacteria (16) | | *L. hongkongensis* | 16 |  | | | | *M. kansasii* | | | | | 9 | | | |  | | | | *R. erythropolis* | | 8 | |
| Lactobacillus (11) | | *L. jensenii* | 3 | | Legionella (36) | | *L. drancourtii* | 7 | Salmonella (14) | | | | *S. enterica* | | | | | 14 | | | | Stenotrophomonas (92) | | | | *S. maltophilia* | | 53 | |
|  | | *L. buchneri* | 2 | |  | | *L. pneumophila* | 18 | Vibrio (43) | | | | *V. cholerae* | | | | | 7 | | | | Micrococcus (16) | | | | *M. luteus* | | 16 | |
|  | | *L. fermentum* | 3 | | Leptospirillum (10) | | *L. ferrodiazotrophum* | 8 |  | | | | *V. metschnikovii* | | | | | 5 | | | |  | | | |  | |  | |
| Parabacteroides ( 5) | | *P. merdae* | 3 | |  | | *L. rubarum* | 7 | Yersinia (26) | | | | *Y. pseudotuberculosis* | | | | | 3 | | | |  | | | |  | |  | |
| Prevotella (19) | | *P. ruminicola* | 1 | | Parachlamydia (31) | | *P. acanthamoebae* | 31 |  | | | | *Y. ruckeri* | | | | | 4 | | | |  |  | | |  | |  | |
|  | | *P. buccae* | 4 | | Ralstonia (212) | | *R. eutropha* | 110 |  | | | | Y. kirkensenii | | | | | 7 | | | |  |  | | |  | |  | |
|  | | *P. denticola* | 4 | |  | | *R. pickettii* | 19 |  | | | |  | | | | |  | | | |  |  | | |  | |  | |
|  | | *P. disiens* | 4 | |  | | *R. solanacearum* | 69 |  | | | |  | | | | |  | | | |  |  | | |  | |  | |
| Roseburia (42) | | *R. intestinalis* | 1 | | Rhizobium (167) | | *R. etli* | 144 |  | | | |  | | | | |  | | | |  |  | | |  | |  | |
|  | | *Roseburia* sp. | 41 | |  | | *R. leguminosarum* | 123 |  | | | |  | | | | |  | | | |  |  | | |  | |  | |
| Ruminococcus (7) | | *R. albus* | 2 | | Streptomyces (449) | | *S. bingchenggensis* | 26 |  | | | |  | | | | |  | | | |  |  | | |  | |  | |
|  | | Ruminococcus sp. | 4 | |  | | *S. himastatinicus* | 37 |  | | | |  | |  | | |  | | | |  |  | | |  | |  | |
| Serratia (14) | | *S. odorifera* | 8 | |  | | *S. pristinaespiralis* | 19 |  |  | |  | |  | |  | | |  | |  | | |  | | |  | |  |
|  | | *S. proteamaculans* | 6 | |  | | *S. scabiei* | 27 |  |  | |  | |  | |  | | |  | |  | | |  | | |  | |  |
| Streptococcus (8) | | *S. agalactiae* | 3 | |  | | *S. violaceusniger* | 26 |  |  | |  | |  | |  | | |  | |  | | |  | | |  | |  |

^#^Metagenomic reads were blasted against the NCBI protein database using a translated nucleotide query. Underrepresented taxonomic genera are not shown.

**Table S2.** **Number and size of contigs that were assigned to (A) *Bacteroides* spp. and (B) to bacterial pathogens associated to the coprolite sample#.**

| **A)Conti ID** | **Contig length (bp)** | **Hit description** | **E-value** | **Hit accession ID** | **Percent ID** |
| --- | --- | --- | --- | --- | --- |
| **contig00127°** | 555 | TonB-dependent receptor [***Bacteroides finegoldii***] | 3E-13 | WP_017142870.1 | 37% |
| **contig00570*** | 195 | ligand-gated channel protein [***Bacteroides coprosuis***] | 5 E-05 | WP_006744840.1 | 45% |
| **contig01043*** | 198 | GTPase HflX [***Bacteroides coprosuis***] | 1E-08 | WP_006744031.1 | 72% |
| **contig02059°** | 291 | collagen-binding protein [***Bacteroides vulgatus***] | 1E-17 | WP_005850005.1 | 65% |
| **contig01426°** | 576 | bacterial group 2 Ig-like protein [***Bacteroides coprocola* CAG:162**] | 9E-17 | CDA71009.1 | 36% |

| B)Conti ID | Contig length (bp) | Hit description | E-value | Hit accession ID | Percent ID |
| --- | --- | --- | --- | --- | --- |
| contig03375 | 504 | hypothetical protein BPP0674 [***Bordetella parapertussis* 12822**] | 1e-32 | NP_883015.1 | 52% |
| contig03491 | 933 | hydrolase [***Bordetella bronchiseptica* 253**] | 2e-72 | YP_006966397.1 | 53% |
| contig02527 | 503 | methyltransferase [***Coxiella burnetii* RSA 493**] | 2e-65 | NP_819712.1 | 68% |
| contig01914 | 1,568 | Possible toxin VapC46. Contains PIN domain [***Mycobacterium tuberculosis* H37Rv**] | 8e-08 | NP_217901.1 | 42% |
| contig03497 | 497 | putative transmembrane protein [***Mycobacterium abscessus***] | 2e-10 | WP_005102565.1 | 30% |
| contig00341 | 529 | hypothetical protein [***Brucella abortus***] | 3e-62 | WP_006089712.1 | 80% |
| contig00638 | 652 | Initiation factor 3 [***Brucella abortus* S19**] and | 1e-24 | YP_001935939.1 | 79% |
| contig01163 | 1,103 | hypothetical protein bgla_1g34580 [***Burkholderia gladioli* BSR3**] | 5e-37 | YP_004362017.1 | 42% |
| contig01000 | 1,019 | hypothetical protein BTI_2181 [***Burkholderia thailandensis* MSMB121**] | 1e-78 | YP_007918661.1 | 70% |
| contig02486 | 704 | paraquat-inducible protein B [***Granulibacter bethesdensis* CGDNIH1**] | 3e-33 | YP_744011.1 | 49% |
| contig01542 | 525 | cation efflux transporter [***Legionella pneumophila subsp. pneumophila***] | 6e-06 | YP_006506440.1 | 45% |
| contig01760 | 833 | Fic/DOC family protein [***Leptospira borgpetersenii***] | 4e-75 | WP_002734514.1 | 52% |
| contig02804 | 1,059 | perosamine synthetase [***Clostridium botulinum***] | 6e-22 | WP_003367053.1 | 70% |
| contig03148 | 808 | methionyl-tRNA formyltransferase [***Clostridium botulinum* A3 str. Loch Maree**] | 1e-34 | YP_001788030.1 | 36% |
| contig01373 | 1,031 | 6-carboxy-5,6,7,8-tetrahydropterin synthase [***Vibrio cholerae***] | 2e-37 | WP_000980008.1 | 75% |
| contig01064 | 537 | hypothetical protein [***Legionella drancourtii***] | 2e-27 | WP_006872680.1 | 40% |
| contig00846 | 1,349 | CP4-6 prophage [***Yersinia kristensenii***] | 2e-124 | WP_004390432.1 | 63% |
| contig01361 | 976 | DNA methylase N-4/N-6 domain protein [***Yersinia kristensenii***] | 3e-140 | WP_004390429.1 | 65% |
| contig03337 | 847 | regulatory protein ada [***Parachlamydia acanthamoebae* UV-7**] | 5e-68 | YP_004652478.1 | 64% |

^#^The contig identifier, its length (bp) and the annotation according to the best BLAST hit (BLASTX *versus* the non-redundant NCBI database, E-value<1e^-05^) are summarized. The E-value, the hit accession identifier and the percent of identity are also provided. Contigs of °human and *pig gut microbiota *Bacteroides* species*.*

**Table S3.** Cultured microorganisms**^#^**

| **Culture condition** | **Cultured microorganism** | **Mode of identification** | **Present in high-throughput pyrosequencing dataset** |
| --- | --- | --- | --- |
| **Schaedler/R2A broths** | *Stenotrophomonas maltophilia* | MALDI-TOF | yes |
|  | *Micrococcus luteus* | MALDI-TOF | yes |
|  | *Paenibacillus macerans* | MALDI-TOF | no |
|  | *Bacillus jeotgali* | MALDI-TOF | yes |
|  | *Staphylococcus pasteuri* | MALDI-TOF | no |
|  | *Staphylococcus epidermidis* | MALDI-TOF | no |
|  | *Staphylococcus cohnii* | MALDI-TOF | no |
|  | *Pseudomonas geniculata* | MALDI-TOF | no |
|  | *Bacillus horti* | 16S rRNA gene sequencing | no |
|  | *Clostridium magnum* | 16S rRNA gene sequencing | no |
|  |  |  |  |
| **Sheep blood culture bottles** | *Paenibacillus macerans* | MALDI-TOF | yes |
|  | *Paenibacillus thiaminolyticus* | MALDI-TOF | no |
|  | *Enterobacter cloacae* | MALDI-TOF | yes |
|  | *Staphylococcus arlettae* | MALDI-TOF | no |
|  | *Propionibacterium acnes* | MALDI-TOF | no |
|  | *Paenibacillus ehimensis* | MALDI-TOF | no |
|  | *Paenibacillus* sp. | 16S rRNA gene sequencing | no |
|  | *Rhodanobacter* sp. | 16S rRNA gene sequencing | no |

**^#^** Reported are the culture conditions, the cultured microorganisms and the mode leading to species identification. Reported is also if the cultured bacteria were found in the high-throughput pyrosequencing dataset. ° When the identification was based on BLAST annotation of the amplified 16S rRNA gene region additional phylogenetic trees were constructed (**Figures S2**).

**Table S4**. **Bacterial pathogens identified from the amplified 16S rRNA V6 region^#^.**

| **Genus** | **Species** | **Reads** | **Size (bp)** | **Reads > 230 bp** |
| --- | --- | --- | --- | --- |
| *Mycobacterium* | *M. kumamotonense* | 1 | 240 | 1 |
| *Bartonella* | *B. henselae* | 47 | 203-251 | 19 |
|  | *B. quintana* | 53 | 270-375 | 53 |
|  | *B. tribocorum* | 1 | 306 | 1 |

**^#^**The species level was defined with a minimum sequence identity of 98.7% using BLAST similarity searches against RDP databases.

**Table S5. Primers used to amplify DNA from intestinal parasites, bacterial pathogens and amoebae.**

| Desired specificity | Gene | Name | Sequence 5'→3' | Tm (C°) | Reference |
| --- | --- | --- | --- | --- | --- |
| *Ascaris* sp. | cytb | Asc1 | GTTAGGTTACCGTCTAGTAAGG | 53 | [[2](#_ENREF_2)] |
|  |  | Asc2 | CACTCAAAAAGGCCAAAGCACC |  |  |
| *Acanthamoeba sp.* | 18S rRNA | IDP1 | TCTCACAAGCTGCTAGGGAGTCA | 60 | [[3](#_ENREF_3)] |
|  |  | IDP2 | TCTCACAAGCTGCTAGGGAGTCA |  |  |
| *Bordetella* sp. | rpoC | F5 | ATGGCGAGGAAGTGCGCCAG | 60 | present study |
|  |  | R5 | CCGTCCGGCTTGGCCATCAG |  |  |
| *Bordetella* sp. | rpoC | F6 | GGCGAGCTCAAGGCCACCAG | 62 | present study |
|  |  | R6 | CAGGTCGGAGGTCGCGAAGC |  |  |
| *Bartonella* sp*.; Brucella* sp. | rRNA_BAA | F7 | GCTGGCGCCCCTGCTTCAAA | 60 | present study |
|  |  | R7 | CCCCGCTGTCTCCAACGCAG |  |  |
| *Bartonella* sp*.; Brucella* sp*.;* | rRNA_rrsC | F8 | TGGCCTGCGATCTATGTTCT | 53 | present study |
|  |  | R8 | AGTCGTAACAAGGTAGCCGT |  |  |
| *Bartonella* sp*.; Brucella* sp.  *Rickettsia* sp.; *Erlichia* sp. | BQ | F12 | ACAAAGGCTAGCGCCCCTGC | 60 | present study |
|  |  | R12 | TCCCCGTGAAGATGCGGGGT |  |  |
|  |  |  |  |  |  |
| *Bartonella henselae* | Spacer | S1 F | TTGCAAAGCAGGTGCTCTCC | 58 | diagnostical tool |
|  |  | S1 R | TAAGCGTGAGGTCGGAGGTT |  |  |
|  | Spacer | S3 F | CAATGGAGGCAACCGTTCTT | 49 | diagnostical tool |
|  |  | S3 R | GTGATATCGGGTACATTTTCAACTG |  |  |
|  | Spacer | S9F | CAACTTCACTGATTTCTGCGATAA | 47 | diagnostical tool |
|  |  | S9R | CGAGGAGTGGTTAATATGACAGCT |  |  |
| *Coxiella burnetii* | spacer | Cox 2F | CAACCCTGAATACCCAAGGA | 59 | diagnostical tool |
|  |  | Cox 2R | GAAGCTTCTGATAGGCGGGA |  |  |
|  | spacer | Cox 5F | CAGGAGCAAGCTTGAATGCG | 59 | diagnostical tool |
|  |  | Cox 5R | TGGTATGACAACCCGTCATG |  |  |
|  |  |  |  |  |  |
| *Legionella* sp. | rpoB | RL1 | GATGATATCGATCAYCTDGG | 55 | diagnostical tool |
|  |  | RL2 | TTCVGGCGTTTCAATNGGAC |  |  |
| *Mycobacterium* sp. | IS6110 | ISMtubF | CCTGCGAGCGTAGGCGTCGG | 68 | present study |
|  |  | ISMtubR | CTCGTCCAGCGCCGCTTCGG |  |  |
|  | rpoB | MF | CGACCACTTCGGCAACCG | 60 | diagnostical tool |
|  |  | MR | TCGATCGGGCACATCCGG |  |  |
| *M. kumamotonense* | 16s rRNA | BF3 | GCGGGTTTTCTCGCAG | 50 | present study |
|  |  | BR3 | GCTCTTTACGCCCAGTAATTC |  |  |
| *M. tuberculosis* | MTC4 | MTC4F | ATGGGTTCGCCAGACGGCGAG | 60 | diagnostical tool |
|  |  | MTC4R | GATCAGCTACGGGTTGGCCG |  |  |
| *Rickettsia* sp. | dksA | dksA-F | TCCCATAGGTAATTTAGGTGTTTC | 54 | diagnostical tool |
|  |  | dksA-R | TACTACCGCATATCCAATTAAAAA |  |  |
|  | mppA | mppA-F | GCAATTATCGGTCCGAATG | 54 | diagnostical tool |
|  |  | mppA-R | TTTCATTTATTTGTCTCAAAATTCA |  |  |
|  | rpmE | rpmE-F | TTCCGGAAATGTAGTAAATCAATC | 54 | diagnostical tool |
|  |  | rpmE-R | TCAGGTTATGAGCCTGACGA |  |  |
| *Streptococcus* sp. | rpoB | Strepto_F | AARYTIGGMCCTGAAGAAAT | 46 | diagnostical tool |
|  |  | Strepto_R | TGIARTTTRTCATCAAACATGTG |  |  |
| *Vibrio cholerae* | ompW | ompW-tsense | CACCAAGAAGGTGACTTTATTGTG | 48 | [[4](#_ENREF_4)] |
|  |  | ompW-antisense | GGTTTGTCGAATTAGCTTCACC |  |  |
|  | cholera toxin | ctxA-s | TCAGACGGGATTTGTTAGGCACG | 50 | [[5](#_ENREF_5)] |
|  |  | ctxA-a-s | TCTATCTCTGTAGCCCCTATTACG |  |  |
|  | chromosom 1 | O1-F | GTTTCACTGAACAGATGGG | 50 | [[5](#_ENREF_5)] |
|  |  | O1-R | GGTCATCTGTAAGTACAA |  |  |
|  | SodB | SodB-F | AAGACCTCAACTGGCGGTA | 50 | [[6](#_ENREF_6)] |
|  |  | SodB-R | GAAGTGTTAGTGATCGCCAGAGT |  |  |
| *Yersinia pestis* | glpD | glpD-F1 | GGCTAGCCGCCTCAACAAAAACAT | 58 | [[7](#_ENREF_7)] |
|  |  | glpD-R2 | GGTGCCAGTTTCAGTAACAC |  |  |

**Table S6.** **The quantitative real-time PCR systems that were tested.**

| Microorganism | GENE | Name | SEQUENCES 5'→3' |
| --- | --- | --- | --- |
| *Brucella* sp. | IS711 | Brucellad | GCTCGGTTGCCAATATCAATG |
|  | IS711 | Brucellar | GGGTAAAGCGTCGCCAGAAG |
|  |  | Brucellap | 6FAM-AAATCTTCCACCTTGCCCTTGCCATCA |
|  |  |  |  |
| *Brucella abortus* | IS711 | Abortusd | GCGGCTTTTCTATCACGGTATTC |
|  | IS711 | Abortusr | CATGCGCTATGATCTGGTTACG |
|  |  | Abortusp | 6FAM-CGCTCATGCTCGCCAGACTTCAATG |
|  |  |  |  |
| *Brucella melitensis* | IS711 | Melitensisd | AACAAGCGGCACCCCTAAAA |
|  | IS711 | Melitensisr | CATGCGCTATGATCTGGTTACG |
|  |  | Melitensisp | 6FAM-CAGGAGTGTTTCGGCTCAGAATAATCC |
|  |  |  |  |
| *Bartonella quintana* | yopP | B qui 11580F | TAAACCTCGGGGGAAGCAGA |
|  | 1ère intention | B qui 11580R | TTTCGTCCTCAACCCCATCA |
|  |  | B qui 11580P | 6FAM- CGTTGCCGACAAGACGTCCTTGC -TAMRA |
|  |  |  |  |
|  | fabF3 | B qui 05300F | GCTGGCCTTGCTCTTGATGA |
|  | fabF3 | B qui 05300R | GCTACTCTGCGTGCCTTGGA |
|  |  | B qui 05300P | 6FAM- TGCAGCAGGTGGAGGAGAACGTG -TAMRA |
|  |  |  |  |
| *Bartonella grahamii* | badA2 | B_gra_3_F | AGATGGAAAAATCCGCTCCA |
|  | 1ere intention | B_gra_3_R | AGGCAAGGGCAAAGAGCATA |
|  |  | B_gra_3_P | 6FAM- TCCGCAACGAGTTCTGGTGGTCA -TAMRA |
|  |  |  |  |
| *Bordetella pertussis* | Toxine | Toxpert2d | CCTACCAGAGCGAATATCTGGCA |
|  | Toxine | Toxpert2r | GCGTTACCCTGCGGATGTTTT |
|  | Toxine | Toxpert2p | 6FAM-ACCGGCGCATTCCGCCC |
|  |  |  |  |
| *Chlamydia pneumoniae* | omp2 | ChPnMGBd | GATTCGTCGCTAGTGCGGA |
|  | omp2 | ChPnMGBr | GTCTAACCTTCTTCGCTGTCA |
|  | omp2 | ChPnMGB | 6FAM-ACAAAGCCAGCACCTGTTCCT -Mgb |
|  |  |  |  |
| *Chlamydia trachomatis* | unknown | Chlam-tracho_1_F | AGCTCCCAAAGCAACCAGAR |
|  | unknown | Chlam-tracho_1_R | BTGTCGCTGCGTTGGTTTTA |
|  | unknown | Chlam-tracho_1_P | 6FAM-CAACAGCACCACCAGCAGCTGC |
|  |  |  |  |
| *Coxiella burnetii* | IS1111A | IS 1111 0706 F | CAAGAAACGTATCGCTGTGGC |
|  | IS1111A | IS 1111 0706 R | CACAGAGCCACCGTATGAATC |
|  | IS1111A | IS1111 07-06 P | 6FAM- CCGAGTTCGAAACAATGAGGGCTG -TAMRA |
|  |  |  |  |
| *Escherichia coli* | ompG | ECOmpGMGBAluId | GCTGCGCGTGCAAATGCG |
|  | ompG | ECOmpGMGBAluIr | CATGGTCATCGCTTCGGTCT |
|  | ompG | ECOmpGMGB | 6FAM-CATCAGAAACTGAACACCAC -Mgb |
|  |  |  |  |
| *Klebsiella pneumoniae* | β-lactamase | KPCall-2F | CGCCGTGCAATACAGTGATA |
| carbapenemase (KPC) | β-lactamase | KPCall-2R | GCAGAGCCCAGTGTCAGCTT |
|  |  | KPCprobeall-2 | 6FAM- CTCTATCGGCGATACCACGT |
|  |  |  |  |
| New Delhi metallo- | metallo-β-lactamase | NDM1-F | GCGCAACACAGCCTGACTTT |
| beta-lactamase-1 (NDM-1) | metallo-β-lactamase | NDM1-R | CAGCCACCAAAAGCGATGTC |
|  |  | NDM1 | 6FAM- CAACCGCGCCCAACTTTGGC |
|  |  |  |  |
| *Legionella pneumophila* | gyrB | Lpneumo_gyrB_MBF | TGGAACCGGTTTGCATCATA |
|  | gyrB | Lpneumo_gyrB_MBR | CGACAGGAATACCACGRCCA |
|  |  | Lpneumo_gyrB_MBP | FAM- TGAATCCCTGGCAGGTTATTGCAAGG |
|  |  |  |  |
| *Leptospira interrogans* | 16S | Lep16SMGBd | GCGGCGAACGGGTGAGTAA |
|  | 16S | Lep16SMGBr | GGAAAGTTATCCAGACTC |
|  |  | Lep16SMGB | 6FAM-ACGTGGGTAATCTT -Mgb |
|  |  |  |  |
|  | hsp | Lint_hsp_MBF | TTCTCGCTCCCAGAGTAGACA |
|  | hsp | Lint_hsp_MBR | TTTTCCAATTGAACTTGAACGTC |
|  |  | Lint_hsp_MBP | FAM- ACTGGCCGACCTTCCAGGTGTAGA |
|  |  |  |  |
| *Listeria monocytogenes* | hlyQ | hlyQf | CATGGCACCACCAGCATC |
|  | hlyQ | hlyQr | ATCCGCGTGTTTCTTTTCGA |
|  | hlyQ | hlyQp | 6FAM-CGCCTGCAAGTCCTAAGACGCCA |
|  |  |  |  |
| *Mycobacterium tuberculosis* | ITS | ITSd | GGGTGGGGTGTGGTGTTTGA |
|  | ITS | ITSr | CAAGGCATCCACCATGCGC |
|  | ITS | sonde tub | 6FAM-GCTAGCCGGCAGCGTATCCAT |
|  |  |  |  |
| *Rickettsia* sp. | unknown | 1029-F1 | GAMAAATGAATTATATACGCCGCAAA |
|  | DIAGNOSTIC | 1029-R1 | ATTATTKCCAAATATTCGTCCTGTAC |
|  | DIAGNOSTIC | Rick1029_MBP | 6FAM- CGGCAGGTAAGKATGCTACTCAAGATAA |
|  |  |  |  |
|  | gltA (CS) | RKND03_F | GTGAATGAAAGATTACACTATTTAT |
|  | EPIDEMIO | RKND03_R | GTATCTTAGCAATCATTCTAATAGC |
|  | EPIDEMIO | RKND03 P | 6FAM- CTATTATGCTTGCGGCTGTCGGTTC -TAMRA |
|  |  |  |  |
| *Rickettsia prowazekii* | ompB | Rpr_ompB_F | AATGCTCTTGCAGCTGGTTCT |
|  | ompB | Rpr_ompB_R | TCGAGTGCTAATATTTTTGAAGCA |
|  | ompB | Rpr_ompB_P | 6FAM- CGGTGGTGTTAATGCTGCGTTACAACA -TAMRA |
|  |  |  |  |
| *Rickettsia felis* | bioB | R_fel0527_F | ATGTTCGGGCTTCCGGTATG |
|  | bioB | R_fel0527_R | CCGATTCAGCAGGTTCTTCAA |
|  | bioB | R_fel0527_P | 6FAM- GCTGCGGCGGTATTTTAGGAATGGG -TAMRA |
|  |  |  |  |
|  | orfb | orfb-f | CCCTTTTCGTAACGCTTTGCT |
|  | orfb | orfb-r | GGGCTAAACCAGGGAAACCT |
|  | orfb | orfb- p (sonde tamra) | 6-FAM-TGTTCCGGTTTTAACGGCAGATACCCA-TAMRA |
|  |  |  |  |
| Shiga toxine de type 2 | stx2 | stx2F | GTGGCATTAATACTGAATTGTCATCA |
|  | stx2 | stx2R | GCGTAATCCCACGGACTCTTC |
|  | stx2 | stx2P | 6FAM- CGGACCTCTGTATCTGCCTGAAGCGTAAGGGTCCG |
|  |  |  |  |
| *Streptococcus pneumoniae* | systeme poc | plyNd | GCGATAGCTTTCTCCAAGTGG |
|  | systeme poc | plyNR | TTAGCCAACAAATCGTTTACCG |
|  | systeme poc | plyN_P | 6FAM-CCCAGCAATTCAAGTGTTCGCCGA |
|  |  |  |  |
|  | lytA | Pneumo_lytA_F | CCTGTAGCCATTTCGCCTGA |
|  | lytA | Pneumo_lytA_R | GACCGCTGGAGGAAGCACA |
|  | lytA | LytA_P | 6FAM-AGACGGCAACTGGTACTGGTTCGACAA |

**Figure S1. Working overview of the polyphasic approach used to analyze the Namur coprolite.**

**
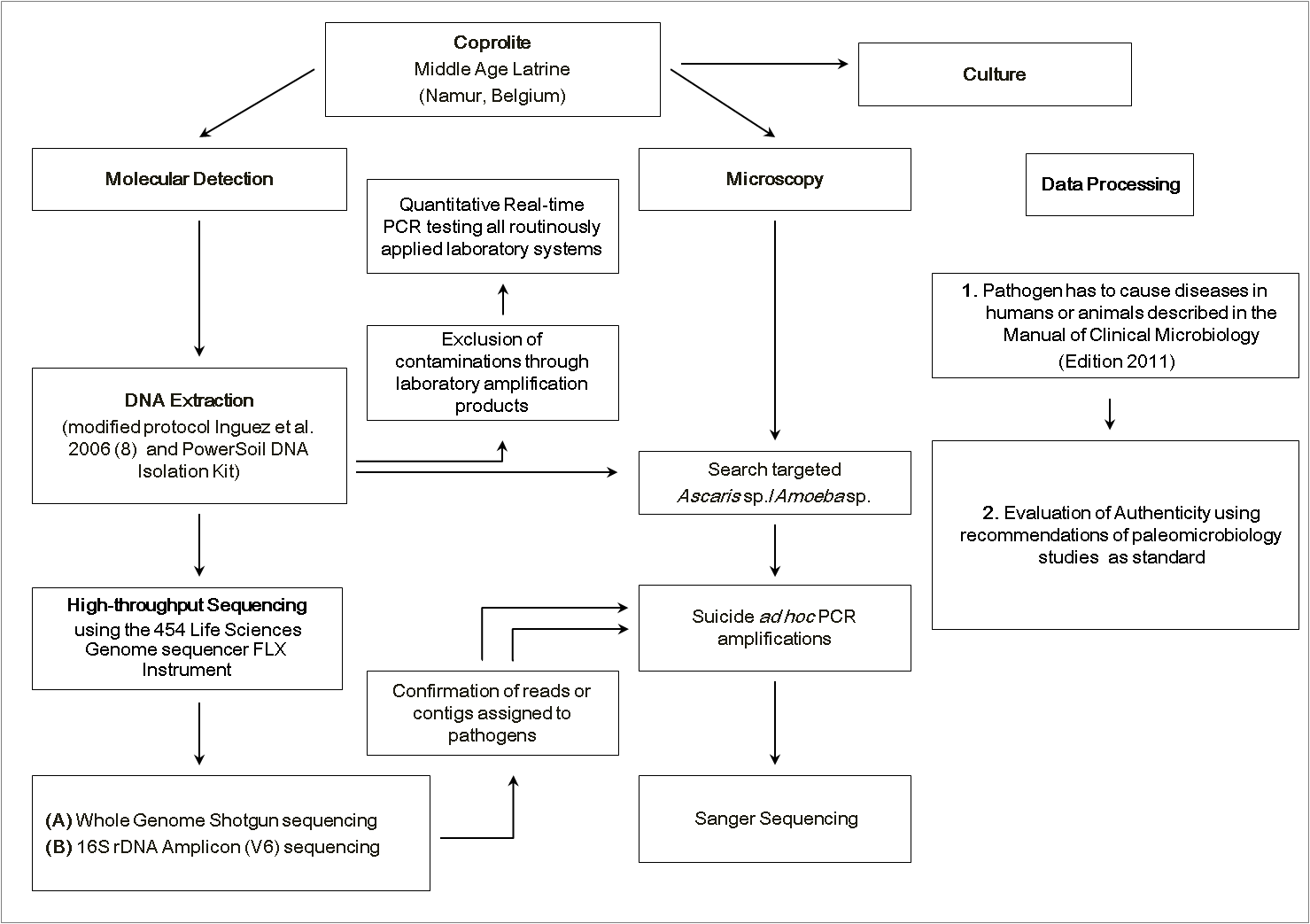
**

**Figure S2.** **Phylogenetic of 16S rRNA gene sequences generated form cultivated bacteria species.** The tree was constructed using the PhyML algorithm with a bootstrap of 100. The bootstrap support is reported for each branch. Phylogenetic tree of 16S rDNA amplicons closely related to (**A**) *Bacillus horti,* (**B**) *Paenibacillus* spp., (**C**) *Rhodanobacter* spp. and (**D**) *Clostridium magnum*.
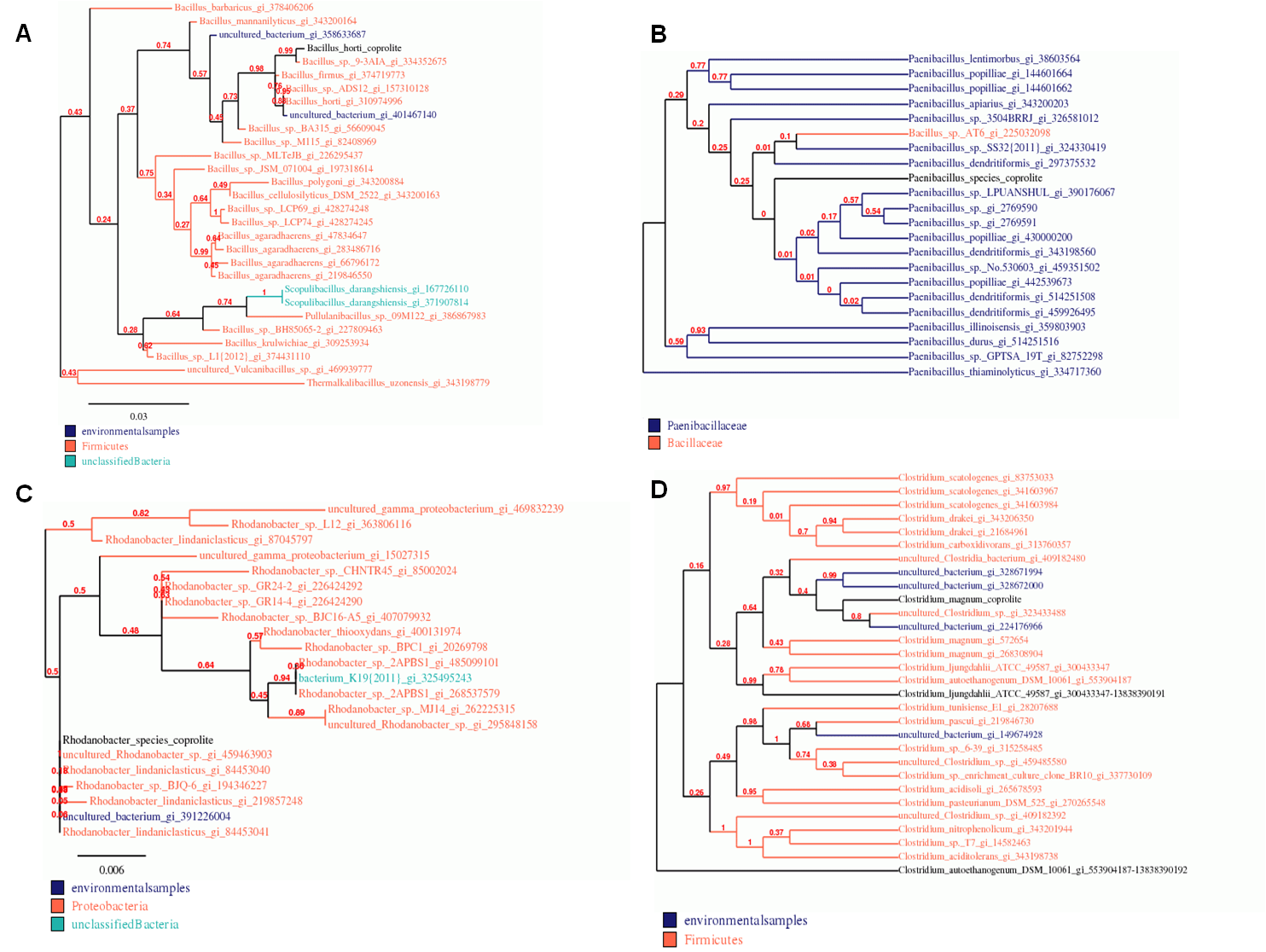


**Figure S3. Phylogenetic tree of a hydrolase.**

A phylogenetic tree was generated from the translated open reading frame of a contig encoding a hydrolase close to *Bordetella* species. The tree was constructed using the PhyML algorithm with a bootstrap of 100. The bootstrap support is reported for each branch.

**
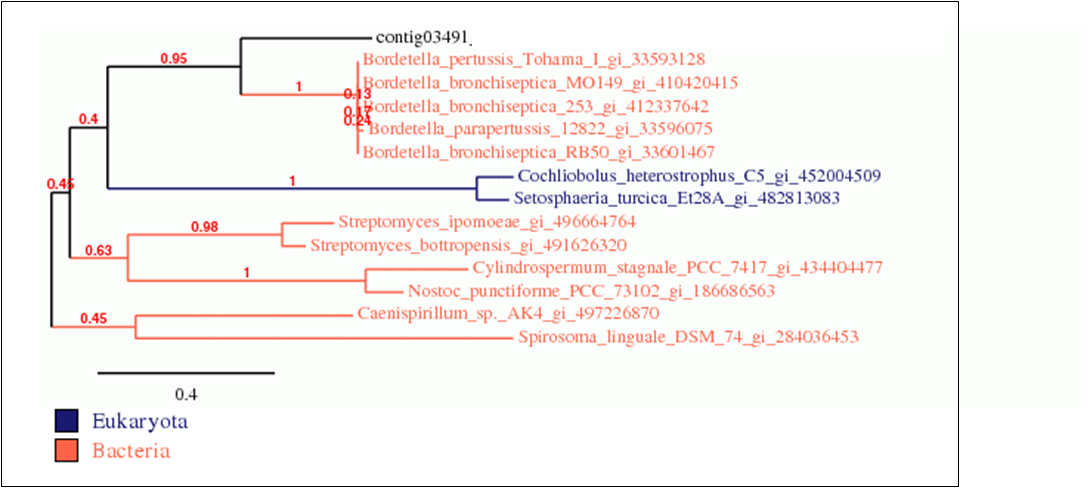
**

**Figure S4. Bayesian source-tracking results.**

(**A**) The mixture of taxa associated to the 16S rDNA gene amplicon dataset of the coprolite specimen was compared to known dataset of various environments. To control the workflow used to perform the analyses, two known samples (**B**) one coprolite previously investigated [[9](#_ENREF_9)] and (**C**) a soil sample [[10](#_ENREF_10)] were positively tested.


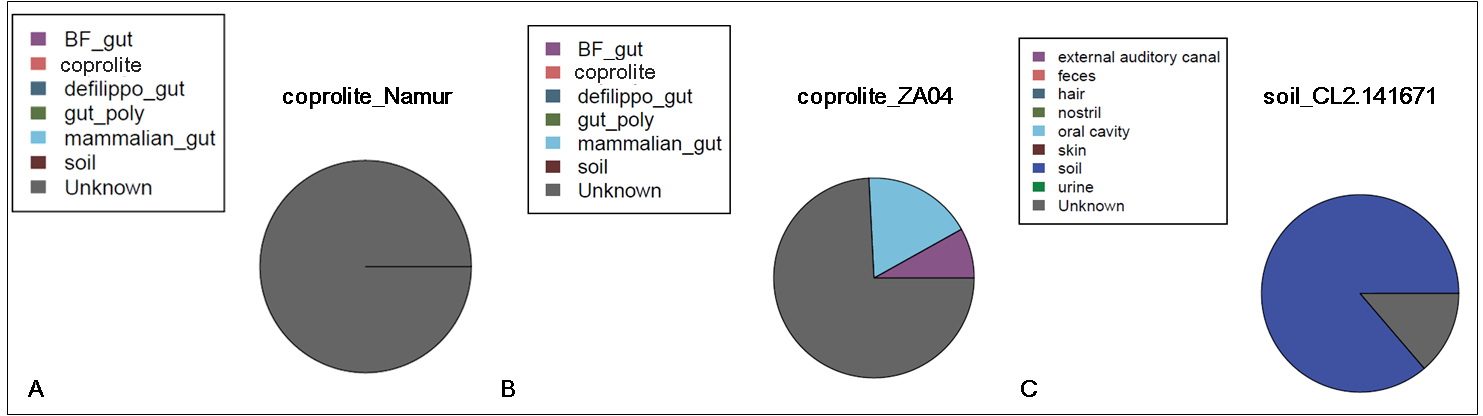


**
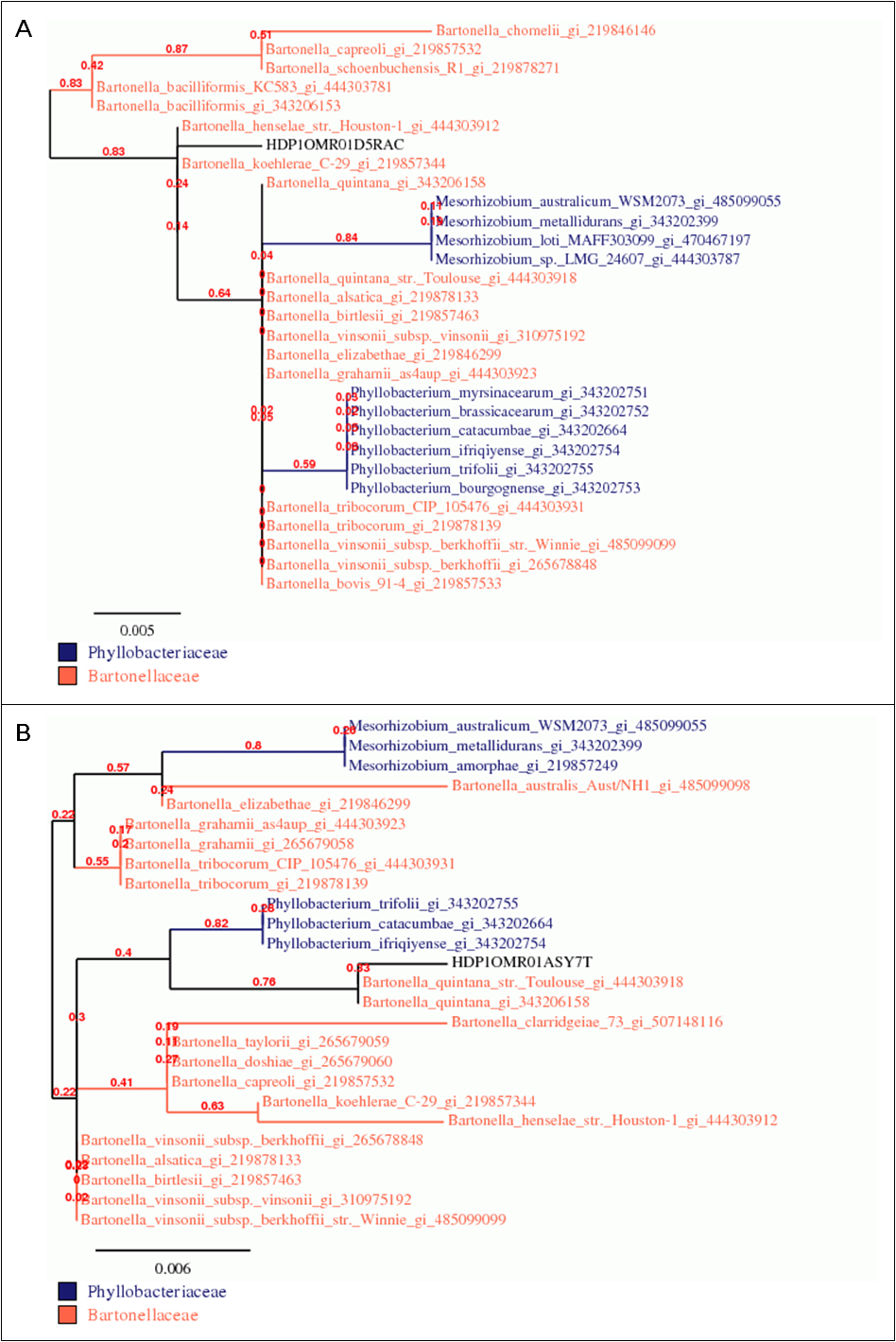
Figure S5. Phylogenetic tree of 16S rDNA amplicons matching to *Bartonella sp.*.**

The tree was constructed using the PhyML algorithm with a bootstrap of 100. The bootstraps are reported for each branch. Phylogenetic tree of 16S rDNA amplicons closely related to (**A**) *B. henselae*, *B. koehlerae* and (**B**) *B. quintana*.

**Figure S6. Alignment and of the amplicon matching to *Bordetella* and *Achromobacter*.**

The sequence alignment was performed using CLUSTALW multiple alignment tool [[11](#_ENREF_11)].


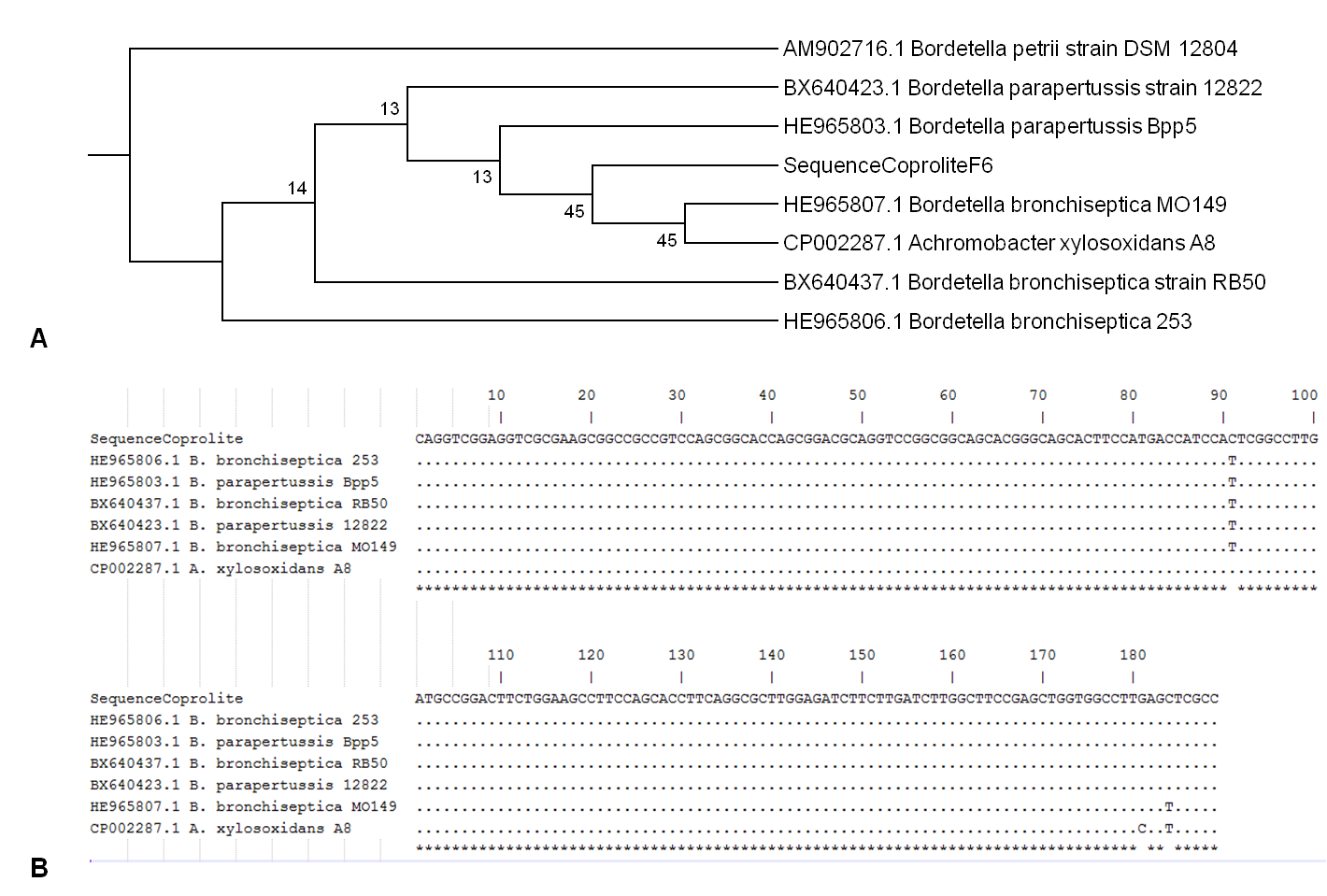


**Figure S7.** **Metabolic comparison of modern metagenomes to the coprolite metagenome.** The Principal coordinates analysis was based on read classification according to BLASTX searches against the SEED Database. For each metagenome included the MG-RAST accession number is given. Compared metagenomes are from soil (yellow cluster), healthy mammalian and human feces (blue cluster); and the coprolite (red). The coprolite metagenome does not group with either the modern gut or soil microbiota.

**Figure A4: Alignment of the amplicon matching to *Bordetella* and *Achromobacter*.** The multiple sequence alignment of the amplicon with *Bordetella sp.* and *Achromobacter xylosoxidans* was performed using CLUSTALW multiple alignment tool (Gibson et al. 1996).

**
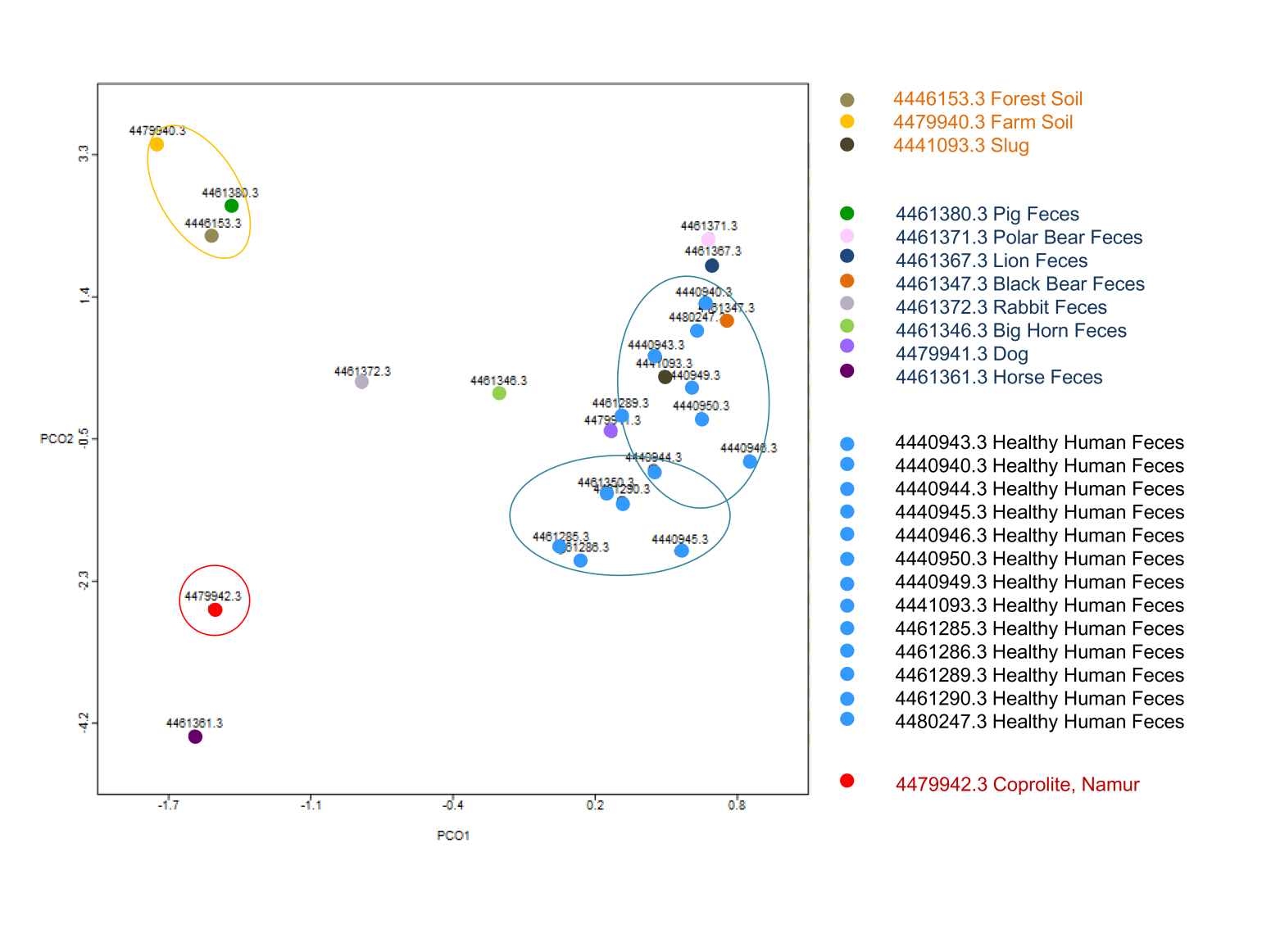
**

**SUPPLEMENTARY REFERENCES**

1. Raoult D, Aboudharam G, Crubezy E, Larrouy G, Ludes B, et al. (2000) Molecular identification by "suicide PCR" of *Yersinia pestis* as the agent of medieval black death. Proc Natl Acad Sci U S A 97: 12800-12803.

2. Loreille O, Roumat E, Verneau O, Bouchet F, Hanni C (2001) Ancient DNA from Ascaris: extraction amplification and sequences from eggs collected in coprolites. Int J Parasitol 31: 1101-1106.

3. Schroeder JM, Booton GC, Hay J, Niszl IA, Seal DV, et al. (2001) Use of subgenic 18S ribosomal DNA PCR and sequencing for genus and genotype identification of acanthamoebae from humans with keratitis and from sewage sludge. J Clin Microbiol 39: 1903-1911.

4. Nandi B, Nandy RK, Mukhopadhyay S, Nair GB, Shimada T, et al. (2000) Rapid method for species-specific identification of *Vibrio cholerae* using primers targeted to the gene of outer membrane protein OmpW. J Clin Microbiol 38: 4145-4151.

5. Lipp EK, Rivera IN, Gil AI, Espeland EM, Choopun N, et al. (2003) Direct detection of *Vibrio cholerae* and ctxA in Peruvian coastal water and plankton by PCR. Appl Environ Microbiol 69: 3676-3680.

6. Tarr CL, Patel JS, Puhr ND, Sowers EG, Bopp CA, et al. (2007) Identification of *Vibrio* isolates by a multiplex PCR assay and rpoB sequence determination. J Clin Microbiol 45: 134-140.

7. Drancourt M, Signoli M, Dang LV, Bizot B, Roux V, et al. (2007) *Yersinia pestis* Orientalis in remains of ancient plague patients. Emerg Infect Dis 13: 332-333.

8. Iniguez AM, Reinhard K, Carvalho Goncalves ML, Ferreira LF, Araujo A, et al. (2006) SL1 RNA gene recovery from *Enterobius vermicularis* ancient DNA in pre-Columbian human coprolites. Int J Parasitol 36: 1419-1425.

9. Tito RY, Knights D, Metcalf J, Obregon-Tito AJ, Cleeland L, et al. (2012) Insights from characterizing extinct human gut microbiomes. PLoS One 7: e51146.

10. Lauber CL, Hamady M, Knight R, Fierer N (2009) Pyrosequencing-based assessment of soil pH as a predictor of soil bacterial community structure at the continental scale. Appl Environ Microbiol 75: 5111-5120.

11. Thompson JD, Higgins DG, Gibson TJ (1994) CLUSTAL W: improving the sensitivity of progressive multiple sequence alignment through sequence weighting, position-specific gap penalties and weight matrix choice. Nucleic Acids Res 22: 4673-4680.
